# LipR functions as an intracellular pH regulator in *Bacillus thuringiensis* under glucose conditions

**DOI:** 10.1002/mlf2.12055

**Published:** 2023-02-11

**Authors:** Xia Cai, Jiaxin Qin, Xuelian Li, Taoxiong Yuan, Bing Yan, Jun Cai

**Affiliations:** ^1^ Department of Microbiology, College of Life Sciences Nankai University Tianjin China; ^2^ School of Life Science and Engineering Lanzhou University of Technology Lanzhou China; ^3^ Key Laboratory of Molecular Microbiology and Technology Ministry of Education Tianjin China; ^4^ Tianjin Key Laboratory of Microbial Functional Genomics Tianjin China

**Keywords:** glucose, intracellular pH, LacI‐type transcription factor, LipR

## Abstract

Intracellular pH critically affects various biological processes, and an appropriate cytoplasmic pH is essential for ensuring bacterial growth. Glucose is the preferred carbon source for most heterotrophs; however, excess glucose often causes the accumulation of acidic metabolites, lowering the intracellular pH and inhibiting bacterial growth. *Bacillus thuringiensis* can effectively cope with glucose‐induced stress; unfortunately, little is known about the regulators involved in this process. Here, we document that the target of the dual‐function sRNA YhfH, the *lipR* gene, encodes a LacI‐family transcription factor LipR as an intracellular pH regulator when *B. thuringiensis* BMB171 is suddenly exposed to glucose. Under glucose conditions, *lipR* deletion leads to early growth arrest by causing a rapid decrease in intracellular pH (~5.4). Then, the direct targets and a binding motif (GAWAWCRWTWTCAT) of LipR were identified based on the electrophoretic mobility shift assay, the DNase‐I footprinting assay, and RNA sequencing, and the *gapN* gene encoding a key enzyme in glycolysis was directly inhibited by LipR. Furthermore, Ni^2+^ is considered a possible effector for LipR. In addition to YhfH, the *lipR* expression was coregulated by itself, CcpA, and AbrB. Our study reveals that LipR plays a balancing role between glucose metabolism and intracellular pH in *B. thuringiensis* subjected to glucose stress.

## Impact statement

Bacterial intracellular pH usually fluctuates according to their growth environment and nutrition. A rapid decrease in the intracellular pH has been observed in yeast and *Escherichia coli* exposed to glucose conditions, and a lower cytoplasmic pH often reduces enzyme activity and inhibits bacterial proliferation. *Bacillus thuringiensis* could commendably cope with excess glucose stress; unfortunately, little is known about the regulators involved in this process. In this study, we discovered that the *lipR* gene encodes a LacI‐family transcription factor LipR as a crucial intracellular pH regulator in *B. thuringiensis* BMB171 exposed to glucose. Our study reveals that LipR, an uncharacterized regulator, plays a balancing role between glucose metabolism and intracellular pH.

## INTRODUCTION

Intracellular pH critically affects many aspects of cell metabolism, such as the activity and stability of enzymes, the rate of biological reactions, the charge of substrates, the structure of different molecules, and signaling processes^1^, ^2^, ^3^. Thus, the intracellular pH is significant for ensuring bacterial growth. Bacteria have evolved to maintain intracellular pH in an optimal physiological range^1^. However, intracellular pH usually fluctuates either slightly or markedly according to the growth environment and nutrition resources^4^.

Glucose is used by many cells as a carbon source, and its catabolism is the backbone of metabolism^5^. Through the glycolytic pathway, glucose is converted into pyruvate, which is then utilized in various metabolic processes. However, excess glucose often leads to the accumulation of acidic metabolites in bacterial cells, for example, acetate, reducing the intracellular pH, impairing enzyme activity, and causing bacterial growth arrest^2^, ^6^, ^7^, ^8^, ^9^. A rapid decrease in the intracellular pH has been observed in yeast and *Escherichia coli* exposed to glucose conditions^2^, ^6^. Unexpectedly, growth arrest resulting from excess glucose was not observed in *Bacillus thuringiensis*. *B. thuringiensis* is a Gram‐positive, rod‐shaped, spore‐forming bacterium and it is the most widely used eco‐friendly bioinsecticide^10^. *B. thuringiensis* exists in sewage, soil, plant roots, insect carcasses, animal intestines, and so forth^11^. Therefore, we sought to determine whether *B. thuringiensis* uses specific regulators to control glucose metabolism in response to glucose stress.

LacI‐family transcription factors (LacI‐TFs) are essential regulators controlling many critical metabolic processes in the cell, such as the metabolism of carbon sources. The most well‐known LacI‐TF is CcpA, a pleiotropic regulator involved in various cellular processes^12^. In the model firmicute *Bacillus subtilis*, CcpA is one of the key players in carbon catabolite repression (CCR). CCR is a regulatory phenomenon in which the utilization of secondary carbon sources is prevented by repressing functional gene expression and reducing the activity of the corresponding enzymes in the presence of preferred carbon sources (e.g., glucose)^13^. In addition, the majority of characterized LacI‐TFs control carbohydrate catabolic pathways and sense sugar effectors^14^, including maltose (MalR)^15^, purines (PurR)^16^, ribose (RbsR)^17^, sucrose (ScrR)^18^, agar (DagR)^19^, and lactose (LacI). Additionally, LacI‐TFs function as the regulators that control the expression of virulence factors (PurR)^20^ and H_2_S production (YcjW)^21^.

Dual‐function sRNAs are a subclass of small regulatory RNAs. On the one hand, they act as base‐pairing sRNAs to modulate target gene expression through noncontiguous base‐pairing with target mRNAs; on the other hand, they function as mRNAs that produce small proteins to participate in the same or another metabolic pathway^22^, ^23^, ^24^, ^25^. To date, only a few dual‐function sRNAs have been characterized, such as RNAIII, Psm‐mec RNA^26^, ^27^, Pel RNA^24^, SgrS^28^, ^29^, AzuCR^30^, Spot 42 RNA^23^, SR1, and SR7^31^, ^32^, ^33^. In our laboratory, we have identified a putative dual‐function sRNA YhfH that is ~500 nucleotides (nt) long and encodes a peptide of unknown function, YhfH‐P, composed of 42 amino acid residues in *B. thuringiensis* BMB171 (data not published). The biological role that YhfH plays in *B. thuringiensis* is still unclear. Therefore, we attempted to dissect the role of YhfH by identifying its target. After analyzing transcriptome data, the *BMB171_C0956* gene located in the complementary strand of the *yhfH* gene was discovered. The *BMB171_C0956* gene encodes a LacI‐TF, and its function is unclear. We hypothesize that the *BMB171_C0956* gene acts as an antisense target of YhfH.

In this study, we sought to determine if the gene *BMB171_C0956* acts as an antisense target of YhfH RNA and to dissect the function of the gene *BMB171_C0956* (named *lipR*). We demonstrate that *lipR* is an antisense target of YhfH and that YhfH represses its expression by influencing mRNA stability. The regulator LipR can tune intracellular pH in the presence of glucose, and its absence would inhibit bacterial growth due to low intracellular pH. Additionally, a binding motif (GAWAWCRWTWTCAT) of LipR was identified, and Ni^2+^ was confirmed as a possible effector for LipR. In addition to YhfH, the *lipR* expression is modulated by the negative regulators LipR and CcpA and the positive regulator AbrB.

## RESULTS

### The regulator LipR is mainly expressed in the stationary phase of cell growth

The *lipR* gene is located in the complementary strand of the *yhfH* gene and encodes a 38.53 kDa protein with a pI of 7.13. To investigate its regulatory function, the expression phase of LipR needs to be determined. First, we determined the transcription start site (TSS) of the *lipR* gene using a 5′‐RACE assay. The results showed that the TSS is an adenine residue (+1) adjacent to the 5′‐RACE adaptor (Figure S1A), and that there is a 33‐bp sequence between the TSS and the start codon GTG of the *lipR* gene. Upstream of the confirmed TSS, the conserved −10 box and −35 box were identified by prediction using the software BPROM, and then the Shine–Dalgarno (SD) (GGGGGAG) was discovered (Figure S1B).

To monitor the expression phase of the *lipR* gene in different environments, we generated the pB‐P*lipR* plasmid by inserting the *lipR* promoter (positions −266 to +16) into the pHT1K plasmid. In the pB‐P*lipR* plasmid, the reporter gene *lacZ* is controlled by the P*lipR* promoter. Then, we measured the β‐galactosidase activity of the strain BMB171/pB‐P*lipR* incubated in Luria–Bertani (LB) (nutrient‐rich) and glucose–yeast–salts (GYS) (nutrient‐poor) media. The results demonstrated that the expression of the *lipR* gene increased significantly from 8 h (exponential phase) to 32 h (stationary phase) and decreased gradually between 36 h and 48 h in the LB medium (Figure S1C). In the GYS medium, *lipR* expression was negligible in the first 6 h, increased significantly from 6 h (exponential phase) to 12 h (stationary phase), and then decreased significantly (Figure S1D). Therefore, the *lipR* gene is mainly expressed in the stationary phase of cell growth, which suggests that the transcription factor LipR primarily plays a regulatory role in the stationary phase of cell growth.

### The *lipR* gene is an antisense target of the dual‐function sRNA YhfH

**Figure 1:**
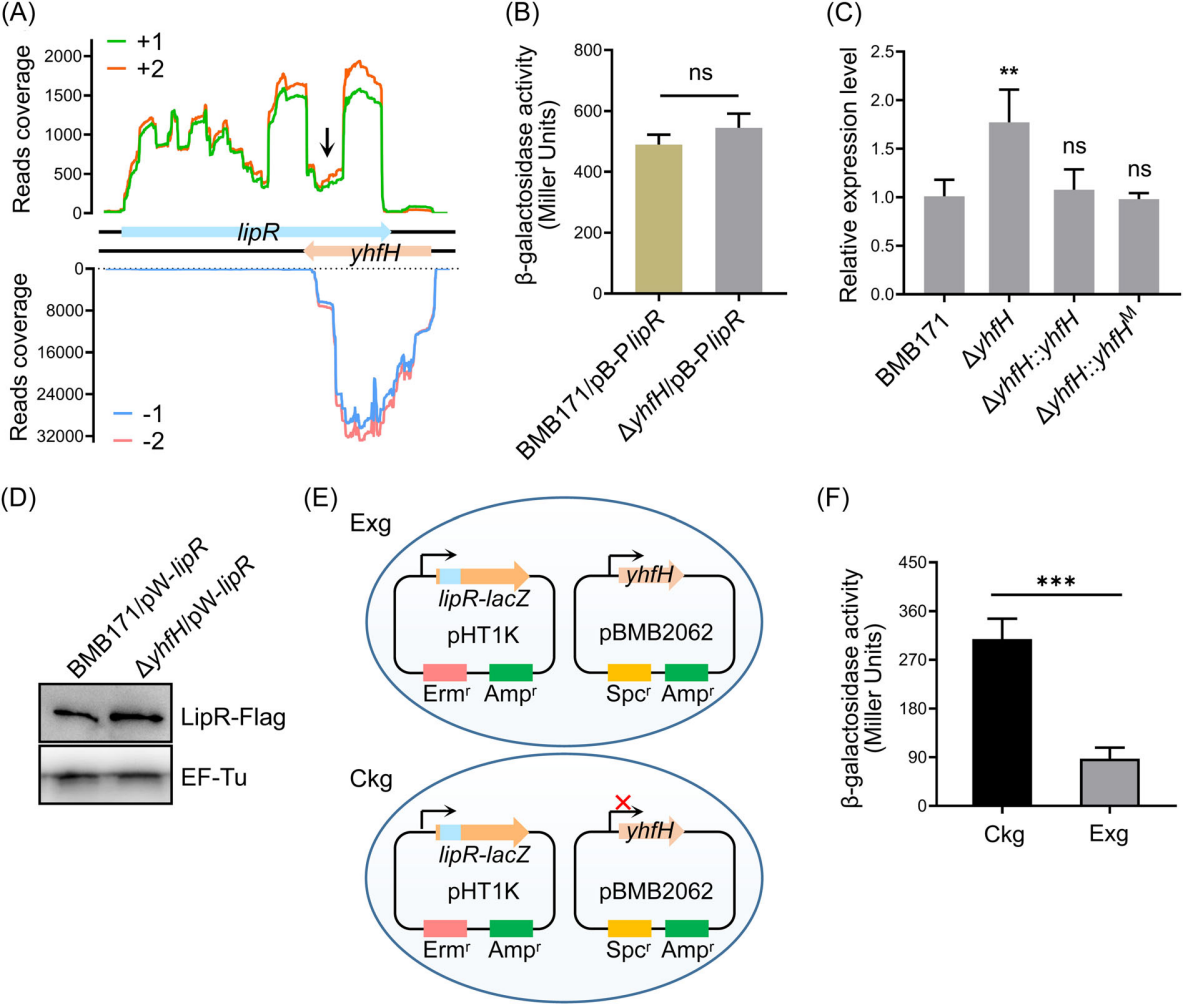
The dual‐function sRNA YhfH acts as an antisense RNA of the *lipR* gene and negatively tunes its expression. (A) Transcriptional landscapes of the genes *lipR* and *yhfH*. 1 and 2 represent two biological replicates; + and – show the sense strand and the antisense strand, respectively. The genes *lipR* and *yhfH* are depicted using light blue and light pink arrows, respectively. The black arrow indicates the region of the *lipR* mRNA where *lipR* mRNA is fully complementary to the YhfH RNA and its transcription level is significantly reduced. (B) Effect of YhfH on the activity of the P*lipR* promoter. The β‐galactosidase activities of strains BMB171/pB‐P*lipR* and Δ*yhfH*/pB‐P*lipR* were detected in GYS medium (9 h). (C) Effect of YhfH RNA on the stability of *lipR* mRNA. The relative expression levels of the *lipR* gene in strains BMB171, Δ*yhfH*, Δ*yhfH*::*yhfH*, and Δ*yhfH*::*yhfH* ^M^ were analyzed using qRT‐PCR. (D) Western blot analysis of the LipR protein fused with a Flag tag in strains BMB171 and Δ*yhfH*. Protein EF‐Tu was used as a loading control^34^, ^35^. (E) Schematic diagram of the construction of the dual‐plasmid system. Plasmids pBMB2062 and pHT1K were used to coexpress YhfH RNA and the partial *lipR* gene (from +691 to +1011) translationally fused inside the ORF of the reporter gene *lacZ* (after 11 codons). The partial *lipR* gene (from +691 to +1011) was translationally fused to the 5′‐region of the *lacZ* ORF to construct plasmid pB‐*lipR*‐*lacZ*, and the entire *yhfH* gene containing its promoter and terminator was cloned into pBMB2062 to generate plasmid pBMB2062‐*yhfH*. Strain Exg carries the plasmids pB‐*lipR*‐*lacZ* and pBMB2062‐*yhfH*, while the control strain Ckg harbors pB‐*lipR*‐*lacZ* and empty plasmid pBMB2062. (F) β‐Galactosidase activities of strains Ckg and Exg. These results were calculated from four biologically independent replicates and are shown as the mean ± SD. **p < 0.01, and ***p < 0.001; ns, no significant difference; GYS, glucose–yeast–salts; ORF, open reading frame; qRT‐PCR, quantitative reverse transcription PCR.

On analyzing the RNA sequencing (RNA‐seq) data of strain BMB171, we discovered a probable antisense RNA YhfH transcribed from the antisense strand of the *lipR* gene, as shown in Figure 1A. YhfH has been considered a putative dual‐function sRNA encoded by the *yhfH* gene. The 3′‐region (~320 nt) of the YhfH RNA and *lipR* mRNA are entirely complementary, and in this region, the transcription level of the *lipR* gene decreased significantly (Figure 1A); thus, YhfH was hypothesized to be an antisense RNA to reduce *lipR* mRNA stability.

To test our hypothesis, the regulatory role of YhfH in *lipR* expression was investigated. The effect of YhfH RNA on the activity of the P*lipR* promoter was tested first. The pB‐P*lipR* plasmid was transformed into strains BMB171 and Δ*yhfH* to measure its activity. The data showed that YhfH RNA did not affect *lipR* transcription by influencing its promoter activity (Figure 1B). Next, the influence of YhfH RNA on *lipR* mRNA stability was verified. The quantitative reverse transcription polymerase chain reaction (qRT‐PCR) results suggested that the mRNA level of the *lipR* gene in the Δ*yhfH* stain was upregulated ~2‐fold compared with that in the BMB171 strain and no significant difference was found compared with the complemented strains Δ*yhfH*::*yhfH* and Δ*yhfH*::*yhfH* ^M^ (Figure 1C). More importantly, strain Δ*yhfH*::*yhfH* ^M^ carries a mutated *yhfH* gene that only encodes the YhfH RNA rather than the peptide product, indicating that the peptide product of the *yhfH* gene was not involved in this regulation. Furthermore, the Western blot results suggested that the protein level of LipR in the Δ*yhfH* strain was higher than that in the BMB171 strain (Figure 1D), which is consistent with the qRT‐PCR results. Thus, antisense RNA YhfH inhibited *lipR* gene expression.

Additionally, a dual‐plasmid system was adapted to confirm that YhfH regulates *lipR* expression in vivo. Two compatible plasmids, pBMB2062 and pHT1K, were used to coexpress YhfH RNA and the partial *lipR* gene (from +691 to +1011) translationally fused to the 5′‐region of the *lacZ* gene (Figure 1E). The results showed that the fused *lipR*‐*lacZ* expression was suppressed when YhfH was overexpressed (Figure 1F), which indicated that YhfH plays an inhibitory role in *lipR* expression, consistent with previous results. The mechanisms by which antisense RNA inhibits target gene expression are by reducing transcript stability (posttranscriptional level) and by preventing mRNA translation (translational level)^36^. On the basis of our data and the large complementary base‐pairing region, YhfH acts as an antisense RNA to modulate the stability of *lipR* mRNA.

### Transcriptome analysis reveals LipR as a regulator of bacterial metabolism

**Figure 2:**
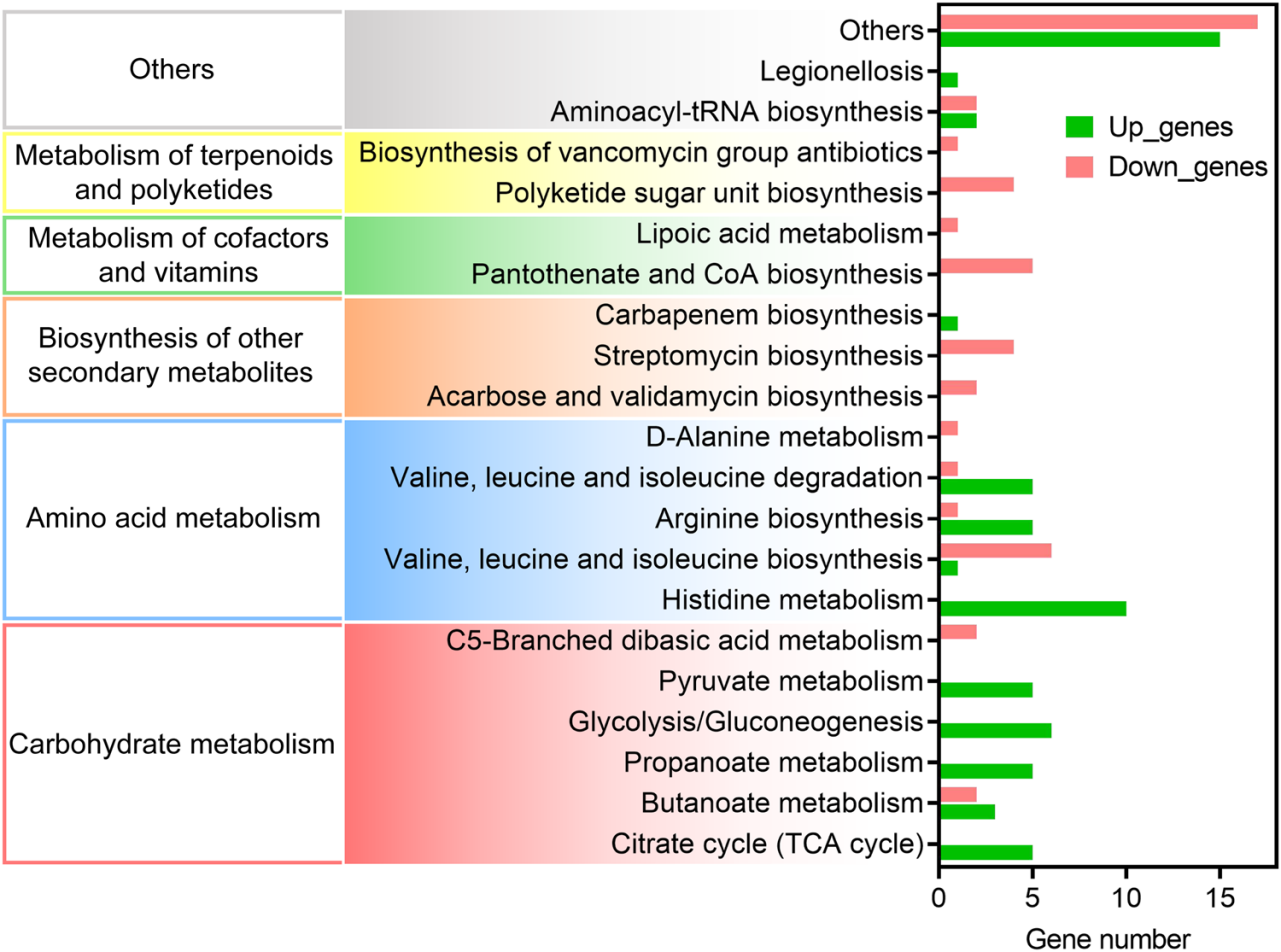
Transcriptome analysis of strains BMB171 and Δ*lipR*. On the basis of the KEGG class, significantly differentially expressed genes between strains BMB171 and Δ*lipR* were classified into different pathways. KEGG, Kyoto Encyclopedia of Genes and Genomes.

An increasing number of LacI‐TFs have been experimentally shown to modulate various bacterial metabolic pathways. To dissect the role of LipR in the cellular processes in *B. thuringiensis*, RNA‐seq was used to analyze gene expression differences between the parent strain BMB171 and mutant Δ*lipR* in an earlier stationary phase (GYS 9 h), a time point at which the *lipR* gene was highly expressed. Comparative transcriptome data revealed that the deletion of the *lipR* gene resulted in 160 differentially expressed genes (|log_2_ FoldChange| > 1, *p* < 0.05), the expression levels of 44 genes were significantly upregulated (Table S2), and 116 genes were significantly downregulated (Table S3). On the basis of the Kyoto Encyclopedia of Genes and Genomes (KEGG) enrichment analysis, these 160 genes were enriched in 20 major cellular pathways. Furthermore, these pathways could be classified into five major metabolic pathways, including amino acid metabolism, carbohydrate metabolism, the metabolism of terpenoids and polyketides, the biosynthesis of other secondary metabolites, and the metabolism of cofactors and vitamins (Figure 2). With the deletion of the *lipR* gene, most of the genes involved in carbohydrate metabolism, the degradation of valine, leucine, and isoleucine, the biosynthesis of arginine, and histidine metabolism were upregulated, and most of the genes participating in the metabolism of terpenoids and polyketides, the biosynthesis of other secondary metabolites, and the metabolism of cofactors and vitamins were downregulated.

### Identification of direct targets and binding sites of LipR

**Figure 3:**
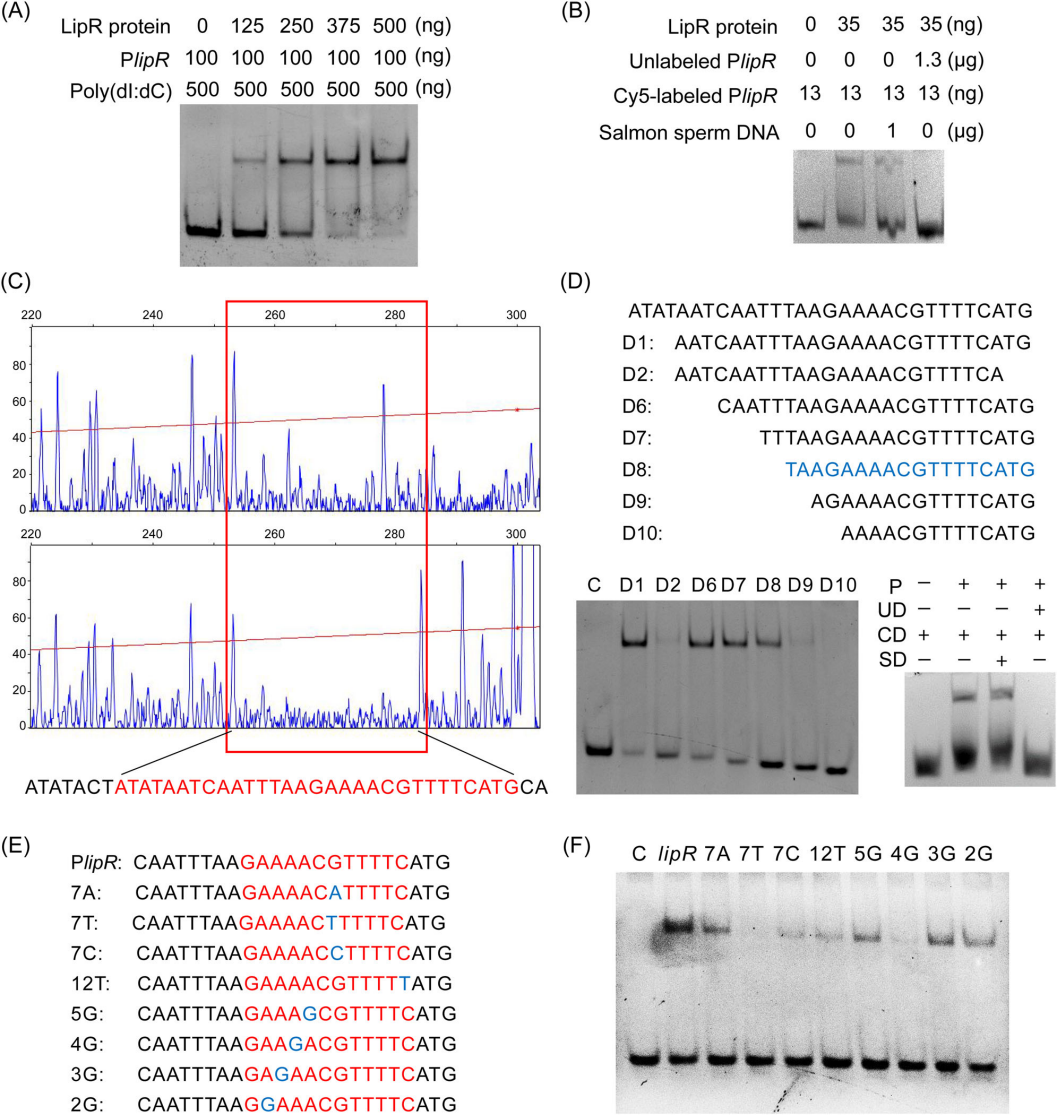
Identification of the precise binding site of LipR embedded in the P*lipR* region. (A, B) The LipR protein directly binds to the promoter P*lipR*. Poly (dI:dC) and salmon sperm DNA were added to prevent nonspecific binding. Unlabeled P*lipR* was used as a specific competitor. Data represent results from three independent experiments. (C) Identification of the DNA region protected by LipR in the P*lipR* promoter. Electropherograms show the 5′‐FAM‐labeled DNA without (upper panel) or with (lower panel) the protection of 0.415 g LipR after DNase‐I digestion. The protected DNA sequence in electropherograms is boxed and shown in red. (D) Determination of the minimum DNA sequence required for binding of the LipR protein by electrophoretic mobility shift assay (EMSA). C, control; CD, Cy5′‐labeled D8 (650 ng); P, LipR protein (35 ng); SD, salmon sperm DNA (1 μg); UD, unlabeled D8 (10 ng). (E) Sequences of DNA fragment D6 carrying various point mutations. (F) EMSA for the LipR protein with different DNA fragments shown in panel (E). Data represent results from three independent experiments. FAM, fluorescein amidites.

Next, we searched for the LipR target using an electrophoretic mobility shift assay (EMSA) combining the RNA‐seq data. Transcription factors are generally self‐regulated^37^. Therefore, His‐tagged LipR was purified, and an EMSA was performed to identify the direct binding between the LipR protein and the P*lipR* promoter. The results demonstrated that the LipR protein binds specifically to the P*lipR* promoter (Figure 3A,B).

**Figure 4:**
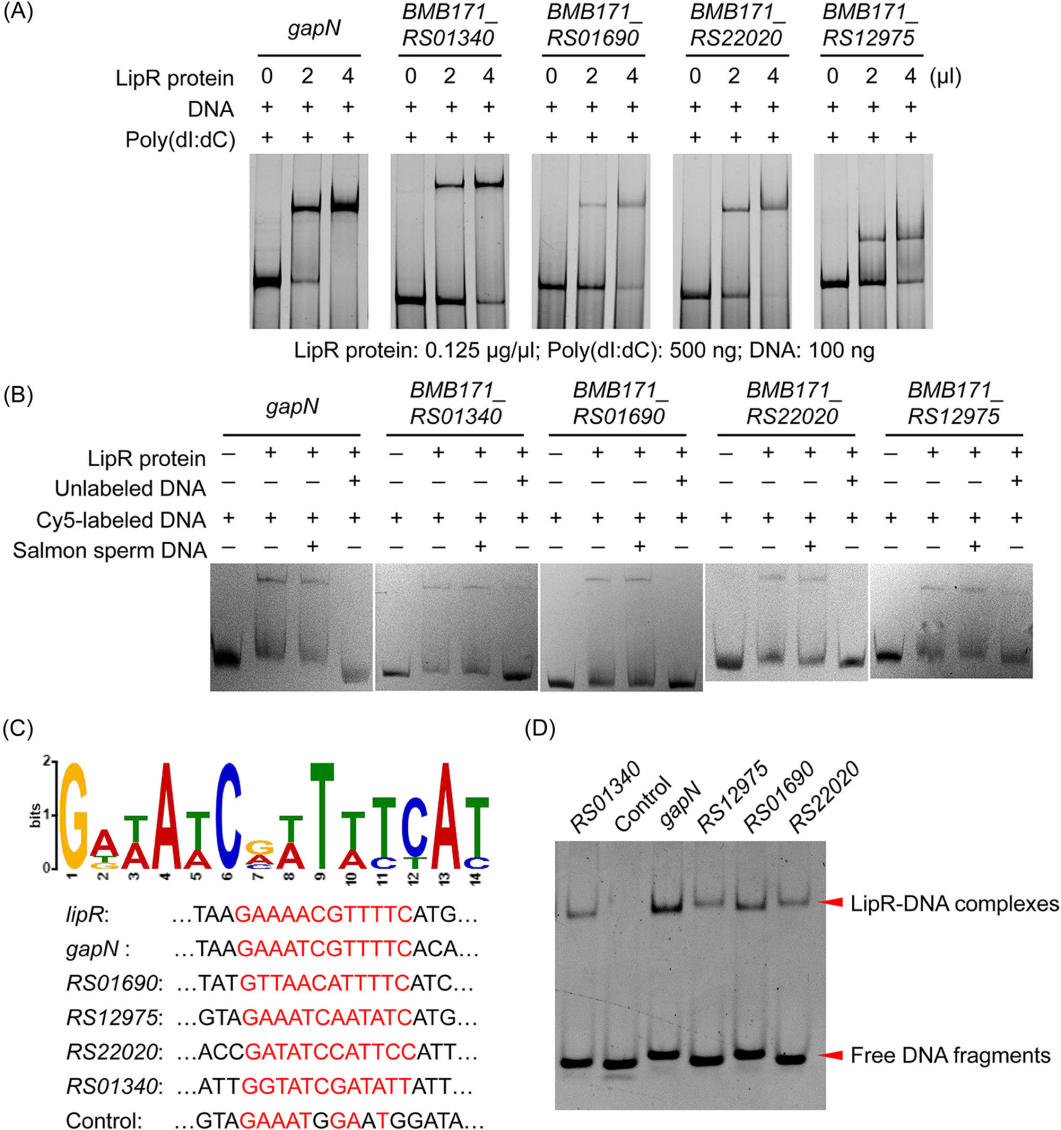
Identification and analysis of the binding motif of the LipR protein. (A, B) Identification of the direct target of LipR by electrophoretic mobility shift assay (EMSA). Poly (dI:dC) was added to prevent nonspecific binding. Salmon sperm DNA (1 μg) was used as a nonspecific competitor; unlabeled P*lipR* (1.3 μg) was used as a specific competitor (B). (C) Potential binding motif of the LipR protein discovered by Multiple Em for Motif Elicitation (MEME) using the P*lipR* promoter and the five obtained direct targets of LipR in panel (A). (D) Binding between LipR and five short DNA fragments tested by EMSA.

Transcription factors commonly bind to sequences located upstream of or adjacent to promoters of their target genes to control their transcription. Therefore, the promoters (350–500 bp) of these 160 differentially expressed genes were amplified to perform EMSA with the LipR protein. We obtained five promoter fragments that could specifically bind with the LipR protein, including the upregulated gene *gapN* and the downregulated genes *BMB171_RS01340* (*BMB171_C0223*), *BMB171_RS01690* (*BMB171_C0264*), *BMB171_RS22020*, and *BMB171_RS12975* (*BMB171_C2358*) (Figure 4A,B). The gene *gapN* encodes glyceraldehyde‐3‐phosphate dehydrogenase (GAPDH), a key enzyme involved in glycolysis. Gene *BMB171_RS01340* encodes a rhomboid family intramembrane serine protease that participates in the degradation of proteins; genes *BMB171_RS01690*, *BMB171_RS22020*, and *BMB171_RS12975* encode uncharacterized proteins (Table S4). According to the direct targets, LipR represses the glycolysis pathway by inhibiting *gapN* expression in the stationary phase of cell growth.

To obtain the precise binding site of LipR, we performed a DNase‐I footprinting assay using the fluorescein amidites (FAM)‐labeled P*lipR* promoter with a size of 300 bp. The results indicated that a 30‐bp sequence appeared to be protected from DNase‐I digestion (Figure 3C), which suggested that the 30‐bp sequence was the precise binding site of LipR. An EMSA experiment using the identified 30‐bp sequence further confirmed this finding (Figure 3D).

To map the minimum binding site of LipR, we utilized EMSA to confirm the binding between the LipR protein and the DNA fragments diminished from 30 bp to 14 bp. Then, we obtained the most streamlined DNA fragment with a size of 18 bp (5′‐TAAGAAAACGTTTTCATG‐3′) that the LipR protein could bind (Figure 3D). Therefore, the 18‐bp sequence is required to recognize and specifically bind to the regulator LipR. Interestingly, it is also a potential candidate target for the global transcription factor CcpA. This implies that CcpA regulates *lipR* expression.

### Analysis of the binding motif of the regulator LipR

The promoters of the five genes identified as direct targets of LipR and the *lipR* promoter were used to search for a common LipR binding motif using Multiple Em for Motif Elicitation software. A conserved 14‐bp motif (GAWAWCRWTWTCAT, where W stands for adenine [A] or thymine [T] and R stands for adenine or guanine [G]) was obtained (Figure 4C).

On the basis of this motif, the specific binding sites of approximately 20–30 bp of LipR upstream of these five genes were scanned and further detected using an EMSA experiment. The results demonstrated that LipR binds directly to these five short fragments (Figure 4D). Interestingly, there were differences in the DNA‐binding affinity between LipR and individual fragments; the binding affinity of LipR on the *gapN* promoter was high, resulting in more protein–DNA complexes than other fragments. The conserved site in the *gapN* promoter is G (+1)‐C(+6)‐G(+7)‐C(+12), that in *BMB171_RS01690* and *BMB171_RS12975* is G (+1)‐C(+6)‐A(+7)‐C(+12), and that in *BMB171_RS22020* is G (+1)‐C(+6)‐C(+7)‐C(+12). Compared with the *gapN* promoter, the G(+7) of the latter three was mutated to A or C, suggesting that the +7 position is G of the LipR binding motif and would facilitate the binding between LipR and the DNA target more than C and A. The conserved site in the promoter of the *BMB171_RS01340* gene is G (+1)‐C(+6)‐G(+7)‐T(+12), and the T in +12 was considered to weaken the binding of LipR to this promoter.

A 23‐bp DNA fragment carrying various point mutations was used to perform an EMSA to further confirm the above conclusion (Figure 3E). The amount of the LipR protein in all reactions was consistent, and then the thickness for the band of the protein–DNA complexes was monitored. Fragment D6 (Figure 3D), a 23‐bp sequence that originated from the P*lipR* promoter, was used as a reference to calculate the binding ability of LipR to other mutants. Consistent with the above conclusion, mutating G (+7) to A or C or T or C (+12) to T in the LipR binding motif reduced the binding of LipR to these sequences. In addition, the positions of +5, +4, +3, or +2 of G weakened the binding affinity of LipR for these sequences, especially for +4 (Figure 3F).

Then, the above motif was used to scan other binding sites of LipR embedded in the BMB171 genome, and the EMSA experiments confirmed partially predicted fragments to which LipR directly binds (Figure S2). In addition to promoters, some binding sites of LipR reside inside genes (Table S5).

### Ion Ni^2+^ is a potential effector of LipR

The binding activity of many LacI‐TFs is affected by one or more specific effectors, and the effectors for them are commonly metal ions, sugars, and the substrate or intermediate of their target genes^14^, ^38^. First, possible effectors for LipR were searched by considering sugars and their metabolites. The *gapN* gene is a direct target of LipR and participates in glycolysis. Thus, sugars (glucose, fructose, xylose, arabinose, galactose, maltose, trehalose, sucrose, cellobiose, mannose, and lactose) and intermediates of glycolysis (fructose‐1,6‐bisphosphate, glucose‐6‐phosphate, pyruvate, and phosphoenolpyruvate) were tested by utilizing EMSA to identify the effector for LipR. The addition of these molecules failed to disrupt the LipR–DNA complexes (Figure S3), which suggested that these molecules are not effectors for LipR. Interestingly, the LipR–DNA complexes were reduced with the addition of pyruvate. This phenomenon probably occurred because this molecule changed the pH in the binding reaction.

**Figure 5:**
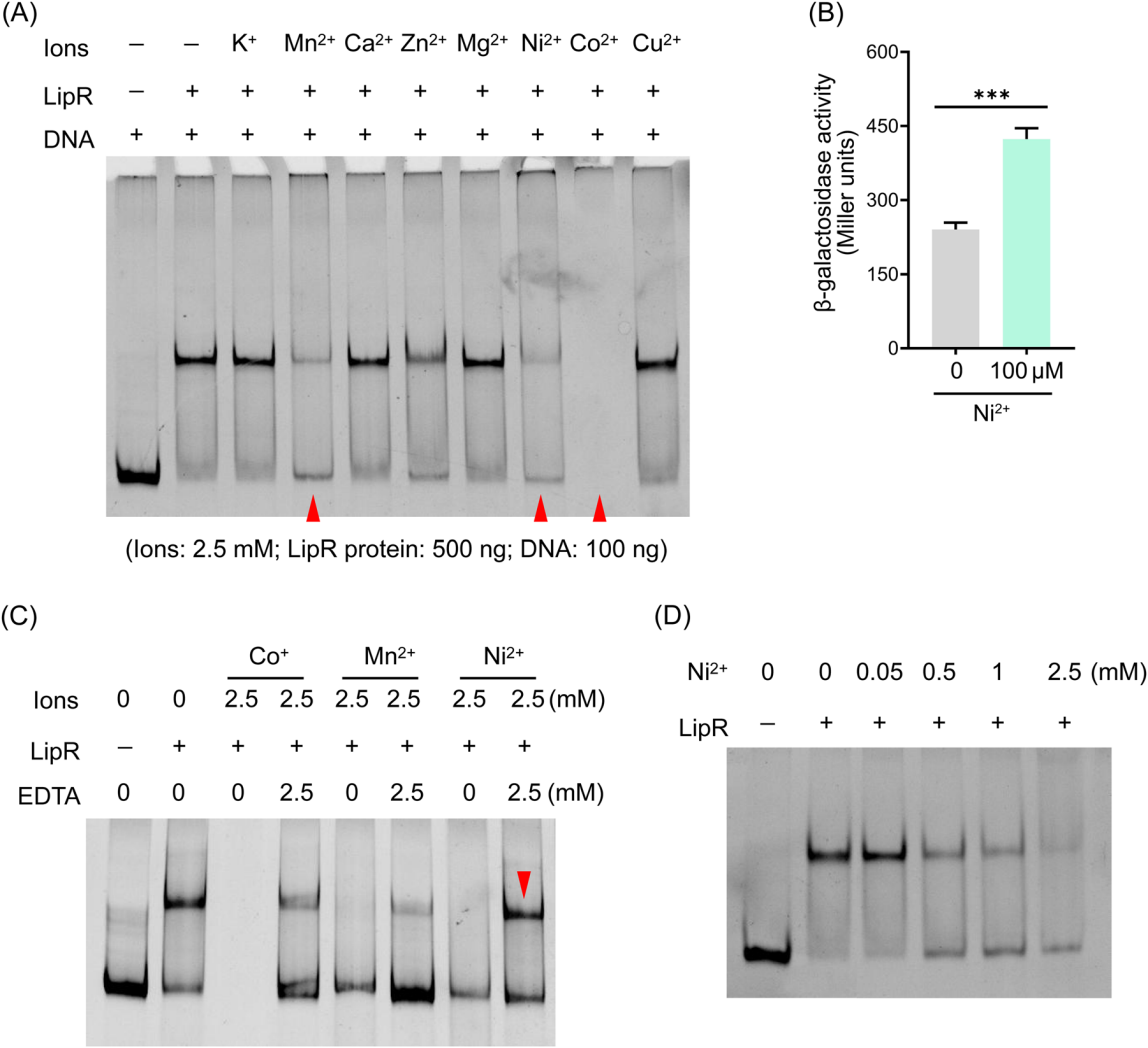
Ni^2+^ is a potential effector for LipR. (A) Influence of eight ions on the binding activity of the LipR protein. With the addition of 2.5 mM Mn^2+^, Ni^2+^, or Co^2+^, the binding activity of the LipR protein was inhibited. The lanes of Mn^2+^, Ni^2+^, and Co^2+^ are marked with red triangles. Data represent results from three independent experiments. (B) Activity of the strain BMB171/pB‐P*lipR* in YS medium supplemented with 0 or 100 μM Ni^2+^. ***p < 0.001. Data represent the mean ± SD from four independent replicates. (C) The LipR protein was denatured after Co^2+^ or Mn^2+^ treatment but not with Ni^2+^ treatment. The LipR protein (0.5 μg) was incubated with 2.5 mM Co^2+^, Mn^2+^, or Ni^2+^ at 28°C for 15 min in alkaline binding buffer A. Then, 0 or 2.5 mM ethylenediaminetetraacetic acid (EDTA) was added to the reaction to chelate the metal ions. After incubation at 28°C for 10 min, the target DNA (0.1 μg) was supplemented to the reaction and incubation was continued at 28°C for 20 min. (D) Increasing concentrations of Ni^2+^ inhibited the binding of the LipR protein to the promoter P*lipR*. Data represent results from three independent experiments. YS, yeast–salts.

It has been reported that metal ions are used as effectors for some TFs^39^. The influence of several metal ions (K^+^, Mn^2+^, Ca^2+^, Ni^2+^, Co^2+^, Zn^2+^, Mg^2+^, or Cu^2+^) on the binding activity for LipR was tested by EMSA. These metal ions were added to the binding reaction at a final concentration of 2.5 mM. EMSA results suggested that the addition of Co^2+^, Mn^2+^, or Ni^2+^ inhibited the formation of LipR–DNA complexes, Zn^2+^ had a weaker effect, and the effect of K^+^, Ca^2+^, Mg^2+^, and Cu^2+^ was negligible (Figure 5A).

To determine whether the presence of ions Co^2+^, Mn^2+^, or Ni^2+^ denatured or allosterically interacted with the LipR protein, the LipR protein was incubated with 2.5 mM Co^2+^, Mn^2+^, or Ni^2+^ at 28°C for 15 min. Then, 2.5 mM ethylenediaminetetraacetic acid (EDTA) was added to the reaction to chelate the metal ions. After 10 min, the target DNA was added to observe whether LipR binds to DNA. The results showed that the LipR–DNA complexes were diminished after Co^2+^ or Mn^2+^ treatment, while the effect of Ni^2+^ was negligible (Figure 5C), implying that the LipR protein was denatured after Co^2+^ or Mn^2+^ treatment but not with Ni^2+^ treatment. This suggests that Ni^2+^ is a potential effector of LipR. Different concentrations of Ni^2+^ were added to the reaction, and the LipR–DNA complexes were reduced as the Ni^2+^ concentration increased (Figure 5D). Then, 0 or 100 μM Ni^2+^ was supplemented in yeast–salts (YS) medium, and the β‐galactosidase activity of strain BMB171/pB‐P*lipR* was determined (Figure 5B). The results showed that the activity of strain BMB171/pB‐P*lipR* was significantly improved with the addition of Ni^2+^. In summary, these data indicated that Ni^2+^ is a potential ligand of LipR.

### Cell growth is specifically inhibited by glucose in the absence of LipR

Stationary‐phase cultures of strains BMB171, Δ*lipR*, and a *lipR* complemented strain Δ*lipR*::*lipR* were diluted in GYS or LB medium to explore whether the absence or presence of LipR influences normal cell growth. The results showed no significant difference in cell growth between these three strains cultured in LB medium (Figure S4A). However, in GYS medium, the cell growth of Δ*lipR* between 0 and 4 h was significantly limited, while strains BMB171 and Δ*lipR*::*lipR* grew much better (Figure S4B). Interestingly, when the late logarithmic phases of cultures of these three strains were diluted in GYS medium, the differences in growth between these three strains were reduced (data not shown). This phenomenon implies that LipR is essential for the environmental transition of bacteria in the stationary phase.

We cultured these three strains in nutrient‐limited medium serum‐supplemented media (SSM) to investigate whether limited nutrients would result in growth differences between the three strains. The data demonstrated that the cell growth of these three strains was almost identical (Figure S4C). The most crucial difference between GYS and SSM media is glucose. Therefore, we suspected that the presence of glucose resulted in a difference in cell growth between these three strains.

**Figure 6:**
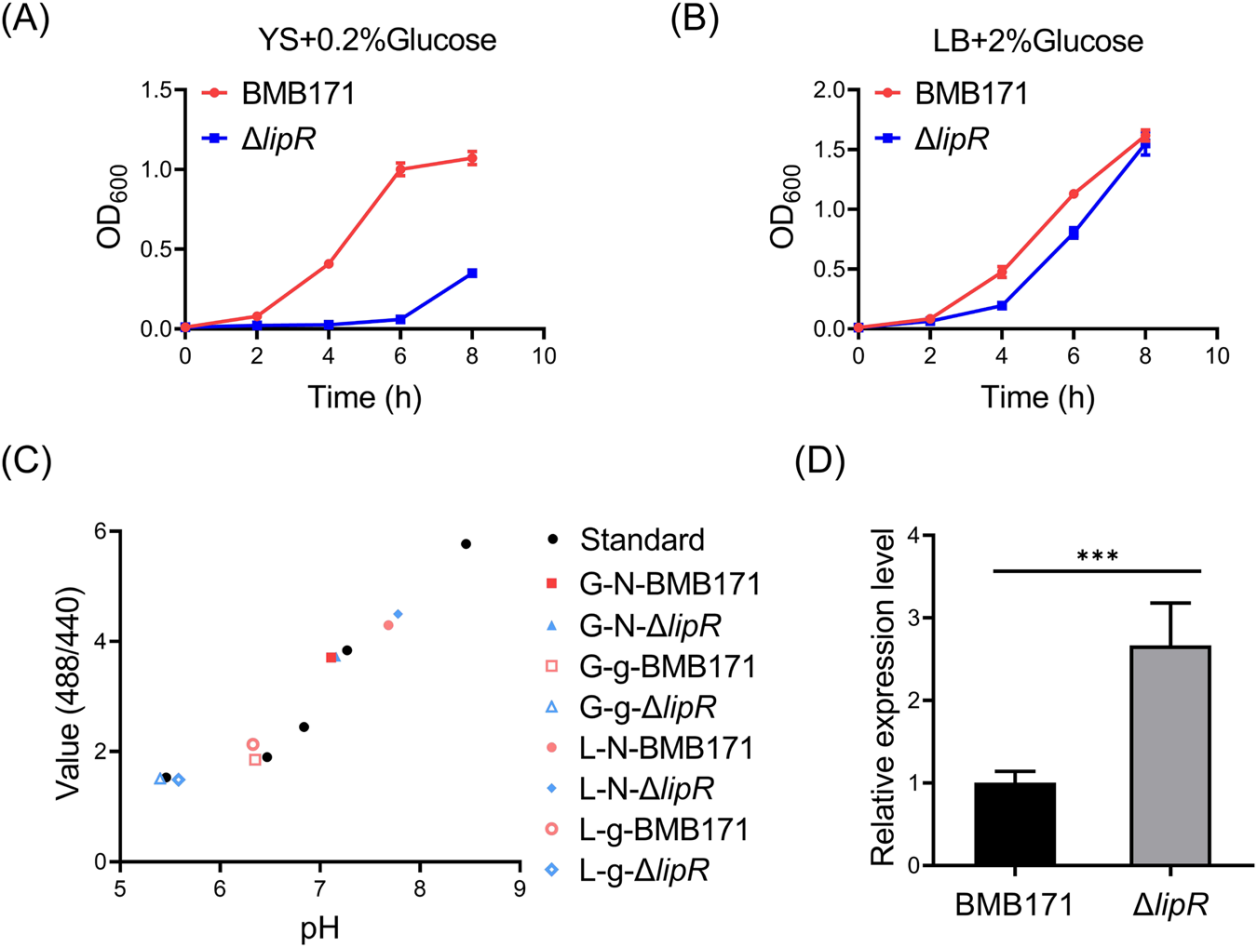
Excess glucose inhibits cell growth of the *lipR* deleted mutant  Δ*lipR* by lowering its intracellular pH. (A, B) The growth of the mutant strain Δ*lipR* is inhibited by glucose. The growth curve of strains BMB171 and Δ*lipR* in YS media with the addition of 0.2% glucose (A) and in LB media supplemented with 2% glucose (B). The results were calculated from four biologically independent replicates and are shown as the mean ± SD. (C) Intracellular pH of strains BMB171 and Δ*lipR* in LB or YS medium with or without glucose determined with the standard dye 2′,7′‐bis‐(2‐carboxyethyl)‐5‐(and‐6)‐carboxyfluorescein, acetoxymethyl ester (BCECF‐AM). BCECF‐AM, a cell membrane‐permeable compound, is widely used to determine the intracellular pH of bacteria. Phosphate buffer with sodium benzoate at different pH was used as standard. The samples were named in the form of X (G or L)‐Y (N or g)‐strain; G, YS medium; L, LB medium; N, no glucose; g, glucose, for example, G‐N‐BMB171, strain BMB171 cultured in YS medium with no glucose; G‐g‐BMB171, strain BMB171 cultured in YS medium with glucose; L‐N‐BMB171, strain BMB171 cultured in LB medium with no glucose; L‐g‐BMB171, strain BMB171 cultured in LB medium with glucose. Data represent results from three independent experiments. The fluorescence intensities of these samples were measured with excitation wavelengths of 440 nm (Exw_440_, pH‐insensitive) or 488 nm (Exw_488_, pH‐sensitive) and emission wavelengths of 535 nm. The *Y*‐axis value shows their ratios of Exw_488_ to Exw_440_ (value [488/440]). (D) Relative expression level of the *gapN* gene in strains BMB171 and Δ*lipR*. ***p < 0.001. The data are shown as the mean ± SD calculated from four biologically independent replicates. LB, Luria–Bertani.

To test our hypothesis, strains BMB171 and Δ*lipR* were incubated in YS medium (glucose‐free GYS medium) supplemented with 0% and 0.2% glucose (m/v), respectively. The growth curves showed no difference in the cell growth of these two strains cultured in YS medium (Figure S4D); however, the cell growth of Δ*lipR* was obviously inhibited with the addition of 0.2% glucose compared with BMB171 (Figure 6A). Moreover, the inhibition phenomenon was also observed by adding 2% glucose to the LB medium (Figure 6B), in which the mutant Δ*lipR* grew slowly, likely due to high glucose.

To investigate whether this phenomenon is explicitly caused by glucose, strains BMB171 and Δ*lipR* were grown in YS medium supplemented with 0.2% galactose, fructose, trehalose, or sucrose. Like glucose, galactose and fructose are monosaccharides with the same relative molecular mass. The only difference among them is the molecular conformation. Interestingly, adding sugars other than glucose did not inhibit the cell growth of strain Δ*lipR* (Figure S4E–H), and the inhibition of cell growth increased with an increase in glucose (Figure S5).

Therefore, the transcription factor LipR is essential for *B. thuringiensis* during the stationary phase when cells are suddenly subjected to increased glucose. The deletion of the *lipR* gene leads to an inhibition of bacterial growth in the presence of glucose.

### Deletion of the *lipR* gene decreases the intracellular pH in BMB171 under glucose conditions

The mechanism by which glucose inhibits the cell growth of strain Δ*lipR* was investigated. In transcriptome data, the absence of LipR leads to upregulation of the *gapN* gene that encodes GAPDH, a key enzyme involved in glycolysis to convert glyceraldehyde‐3‐phosphate into 1,3‐bisphosphoglycerate^5^. Moreover, it has been reported that excess glucose leads to the accumulation of acid metabolites, such as acetate, which reduces the intracellular pH and causes growth arrest in *E. coli* ^6^. Finally, the *gapN* gene is the only gene involved in the glycolysis/gluconeogenesis pathway that is directly regulated by LipR (Table S6). Therefore, we speculated that the higher rate of glycolysis induced by the upregulation of the *gapN* gene leads to a decrease in intracellular pH, thus inhibiting the growth of bacterial cells. The intracellular pH of strains BMB171 and Δ*lipR* was measured under glucose or no‐glucose conditions. The data showed that in the absence of glucose, the intracellular pH of strains Δ*lipR* and BMB171 remained neutral or was slightly higher (G‐N‐Δ*lipR*, L‐N‐Δ*lipR*, G‐N‐BMB171, L‐N‐BMB171); however, the intracellular pH of Δ*lipR* was lower than that of BMB171 in the presence of glucose in both LB and YS media (Figure 6C). We observed that strain Δ*lipR* cultured in LB with 2% glucose (L‐g‐Δ*lipR*) or YS with 0.2% glucose (G‐g‐Δ*lipR*) reduced the intracellular pH to approximately 5.4, while the pH of BMB171 (G‐g‐BMB171 and L‐g‐BMB171) remained above 6.5. Thus, the inhibition of glucose on the growth of strain Δ*lipR* is due to the lower intracellular pH. This result suggests that LipR plays a role in regulating intracellular pH by repressing *gapN* expression when bacteria are suddenly exposed to a glucose environment.

### The *lipR* expression is also regulated by its protein product, CcpA, and AbrB

**Figure 7:**
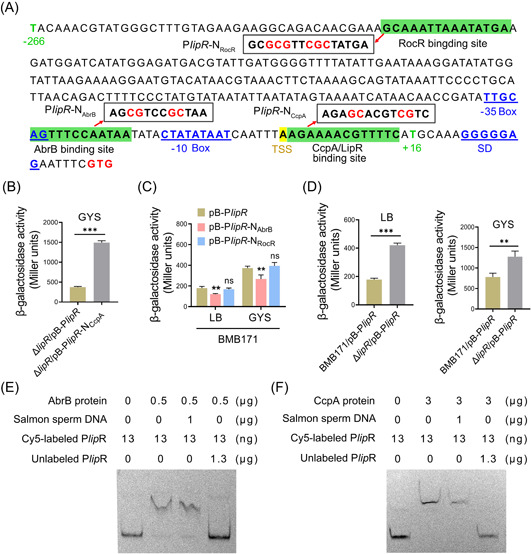
The *lipR* expression is modulated by the positive regulator AbrB and the negative regulators LipR and CcpA. (A) Sequence of the P*lipR* promoter carrying potential RocR, AbrB, and CcpA binding sites. The predicted RocR, AbrB, and CcpA binding sites are shaded in green, and their mutants are boxed in black. (B, C) Regulators CcpA and AbrB modulate the activity of the P*lipR* promoter, and the transcription factor LipR inhibits its activity (D). pB‐P*lipR*, plasmid pHT1K carries the original P*lipR* promoter (for details of the plasmid construction, see the Supporting Information). pB‐P*lipR*‐N_CcpA_, plasmid pHT1K carries a mutated P*lipR* promoter with no CcpA‐recognized motif; pB‐P*lipR*‐N_AbrB_, plasmid pHT1K carries a mutated P*lipR* promoter with no binding site of AbrB; and pB‐P*lipR*‐N_RocR_, plasmid pHT1K carries a mutated P*lipR* promoter with no potential RocR‐recognized site (A). These four plasmids were transformed into strain BMB171 or Δ*lipR* to measure β‐galactosidase activity (B–D). The β‐galactosidase activities of various strains in LB and GYS media were calculated from four biologically independent replicates and are shown as the mean ± SD. **p < 0.01, and ***p < 0.001; ns, no significant difference. (E, F) Direct interactions between the P*lipR* promoter and proteins AbrB and CcpA detected by EMSA. Salmon sperm DNA, for a nonspecific competitor; unlabeled P*lipR*, for a specific competitor.

In addition to the antisense RNA YhfH, we wanted to determine whether other transcription factors were involved in regulating *lipR* expression. Transcription factors are generally self‐regulated, and we have identified that a binding site of LipR is embedded in the P*lipR* promoter (Figure 7A); thus, the self‐regulation phenomenon of the *lipR* gene was investigated. The activities of strains BMB171 and Δ*lipR* carrying plasmid pB‐P*lipR* were measured. The results showed that the activity of the P*lipR* promoter in strain Δ*lipR* was upregulated both in LB (6 h) and GYS (8 h) media (Figure 7D), which indicated that LipR played an inhibitory role in its expression.

Previously, we discovered that a candidate binding site of CcpA resides in the P*lipR* promoter (Figure 7A), suggesting that CcpA might participate in regulating *lipR* expression. To test this, we performed an EMSA to test the binding between the CcpA protein and the P*lipR* promoter with salmon sperm DNA added to reduce nonspecific binding. The results demonstrated that CcpA specifically binds to the P*lipR* promoter (Figure 7F). Furthermore, we carried out an in vivo experiment to dissect the regulatory role of CcpA in *lipR* expression. We created a novel plasmid pB‐P*lipR*‐N_CcpA_ carrying a mutated P*lipR* promoter that changed the binding site of CcpA (Figure 7A). Since the binding site of CcpA is identical to that of LipR, the Δ*lipR* strain was chosen as the host in this experiment. The activity of the Δ*lipR* strain containing the plasmids pB‐P*lipR*‐N_CcpA_ and pB‐P*lipR* was determined in GYS medium (6 h). The data showed that the activity of the promoter P*lipR* was upregulated when the binding site of CcpA was mutated (Figure 7B), indicating that CcpA represses the LipR expression.

In addition, the potential binding site of the regulators AbrB and RocR was discovered in the P*lipR* promoter using the software DBTBS (Figure 7A). AbrB is a global regulator that mainly controls gene expression in the exponential or transition phase^40^. The mutation in the AbrB binding site reduced the activity of the P*lipR* promoter (Figure 7C), demonstrating that AbrB promotes LipR expression. However, mutation in the RocR binding site did not influence the P*lipR* activity, which may be because the potential binding site of RocR is too far away from the P*lipR* promoter (a distance of 175 bp) (Figure 7C). His‐tagged AbrB protein was purified and used to analyze its binding with the P*lipR* promoter. The EMSA results were consistent with the above results, which indicated that AbrB directly binds to the P*lipR* promoter and promotes LipR expression (Figure 7E).

In short, the expression of the *lipR* gene is controlled by itself and the transcription factors CcpA and AbrB. LipR and CcpA suppress the expression of *lipR*, while AbrB promotes it.

### Homologs of the LipR protein are widely distributed among bacteria

**Figure 8:**
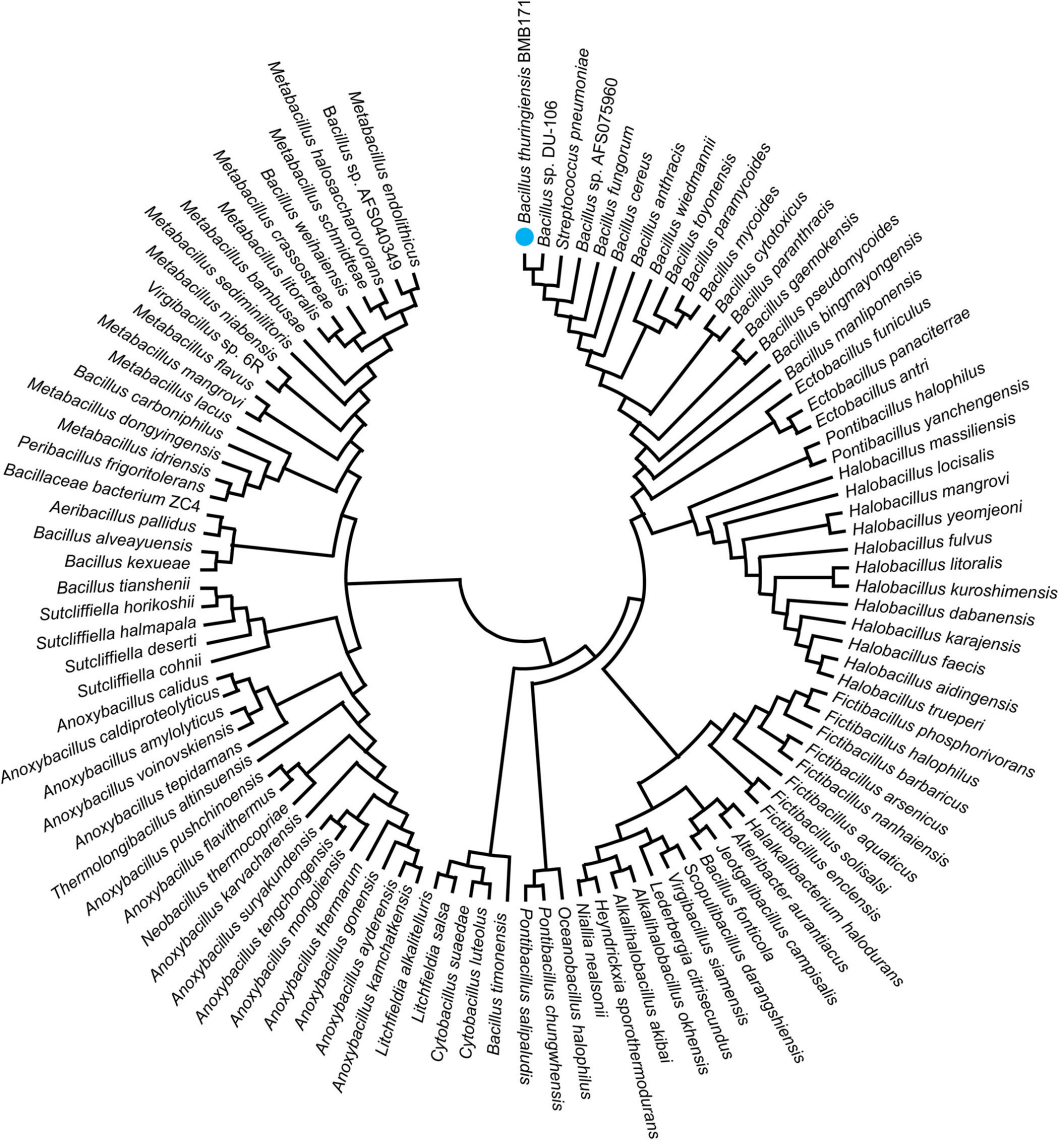
Distribution and phylogenetic tree of the homologs of LipR (Query cover >90%, Identity >50%). The LipR protein that originated from *Bacillus thuringiensis* BMB171 is marked with a blue circle.

The function and regulation of the LipR protein have been investigated in detail, and further research has focused on the distribution and conservation of LipR‐like proteins. Homologs of LipR were searched using BLASTp among *Bacillus cereus* group strains, including the human anthrax pathogen *Bacillus anthracis* and opportunistic pathogen *B. cereus*, and it was discovered that LipR‐like proteins were widely present and highly conserved in the *B. cereus* group, with sequence identities ranging from 90.21% to 99.15% and Query cover of 100%. Next, homologs of LipR were searched among bacteria with an identity higher than 50% and Query cover higher than 90%, and the results showed that LipR‐like proteins are widely present in the groups *Bacillus* sp., *Lysinibacillus* sp., *Ectobacillus* sp., *Anoxybacillus* sp., *Sutcliffiella* sp., *Cytobacillus* sp., *Metabacillus* sp., *Aeribacillus* sp., *Litchfieldia* sp., *Fictibacillus* sp., *Virgibacillus* sp., *Halobacillus* sp., *Pontibacillus* sp., *Oceanobacillus* sp., *Halalkalibacterium* sp., *Lederbergia* sp., *Alkalihalobacillus* sp., *Paenalkalicoccus* sp., *Alkalicoccus* sp., *Heyndrickxia* sp., *Niallia* sp., *Salisediminibacterium* sp., and *Jeotgalibacillus* sp. (Figure 8). This implies that those strains possibly also use LipR‐like proteins to regulate glucose metabolism and intracellular pH.

## DISCUSSION

Most LacI‐TFs have been characterized and documented to control the metabolism of carbon sources. In this study, we demonstrated that a function‐unknown LacI‐TF LipR acted as an intracellular pH regulator in the presence of glucose. The absence of LipR would increase the sensitivity of *B. thuringiensis* to glucose stress. The binding motif of LipR was identified as GAWAWCRWTWTCAT, and Ni^2+^ was shown to be a potential effector for LipR. In addition to dual‐function sRNA YhfH, *lipR* expression was modulated by the negative regulators LipR and CcpA and the positive regulator AbrB.

The dual‐function sRNA YhfH has been proven to be a negative regulator of LipR. Unfortunately, knockout of the *yhfH* gene did not result in a distinct phenotype under glucose exposure (data not shown). We suspect that the slight overexpression (~2‐fold) of LipR induced by the absence of YhfH does not cause a marked change in metabolism; thus, we did not observe a noticeable phenotypic change. This phenomenon also implies that the slight overexpression of LipR in the presence of glucose has no adverse effect on bacterial cells.

CcpA is a global regulator that facilitates the use of preferred carbon sources, such as glucose. Specifically, CcpA inhibits the metabolism of secondary carbon sources in the presence of glucose. Here, CcpA inhibits the expression of the *lipR* gene, and the latter prevents the expression of the *gapN* gene. In other words, CcpA promotes *gapN* expression. Consistent with previous reports, CcpA plays a positive role in glycolysis.

Interestingly, the binding sites of LipR and CcpA embedded in the P*lipR* promoter are identical. The consensus motif of CcpA is WTGNAANCGNWNNCW listed in DBTBS software, and that of LipR is GAWAWCRWTWTCAT. The conserved sites of LipR, G (+1) C (+6) C (+12), are identical to G (+3) C (+8) C (+14) of the CcpA motif. The +9 position of the CcpA motif is G; however, the +7 position of the LipR motif is G or A. When the seventh position in the binding site of the LipR target is G, the target may be regulated by both LipR and CcpA. This phenomenon implies that these two transcription factors have many target genes in common, their functions may be complementary or competitive, and their relationship needs to be further explored. This study will provide a deeper understanding of how bacteria regulate glucose metabolism and intracellular pH.

Excess glucose induces the accumulation of acidic metabolites in bacterial cells, for example, acetate^7^, ^8^, which lowers the intracellular pH and therefore inhibits cell growth^6^, ^9^. In our study, adding glucose lowered the cytoplasmic pH, which resulted in growth inhibition of strain Δ*lipR*. Conversely, the parent strain BMB171 could maintain a suitable intracellular pH (above 6.5) under glucose conditions. This suggests that the regulator LipR plays a critical role in regulating the intracellular pH in *B. thuringiensis* BMB171 under glucose conditions.

Generally, transcription factors govern cellular metabolism by modulating the expression of their target genes with corresponding functions. We have identified six target genes for LipR. Of these, the product GAPDH of the *gapN* gene is directly related to glucose metabolism. Hence, gene *gapN* is considered a key mediator in the regulation of intracellular pH by LipR. GAPDH has been documented to be an essential bottleneck in glycolytic flux^5^, ^41^. Consequently, overproduction of GAPDH caused by the *lipR* deletion leads to increased glycolysis flux and accumulation of acidic metabolites, resulting in a low intracellular pH. Of course, we do not exclude other target genes of LipR or those not identified because the binding site residing in the coding sequence is also involved in maintaining pH.

Ion Ni^2+^ was verified to act as a potential effector for LipR. Bacteria utilize metal ions as cofactors and structural elements for many proteins, such as Cu^2+^, Zn^2+^, Fe^2+^/Fe^3+^, Mn^2+^, Co^2+^, and Ni^2+^ ^42^. Ni^2+^ plays an essential role in catalyzing some biological processes, such as urea hydrolysis, molecular hydrogen consumption, and methane formation^43^. It is worth noting that Ni^2+^ is also toxic for many proteins by replacing their essential metals and binding to them to inhibit their activity allosterically^44^. The binding motifs of Ni^2+^ are H(X)_*n*_H, M(X)_*n*_H, and H(X)_*n*_M (where X stands for any amino acid)^43^. The LipR protein has two M(X)_*n*_H sites and one H(X)_*n*_H site (Figure S6), indicating that it has a structural basis for binding Ni^2+^. Moreover, proteins such as l‐lactate dehydrogenase, dTDP‐4‐dehydrorhamnose reductase, and GAPDH were proven to be Ni^2+^‐binding proteins. In the *B. thuringiensis* strain, GAPDH is encoded by the *gapN* gene, a direct target of LipR. In addition, a binding site of LipR is located in the promoter of the l‐lactate dehydrogenase‐encoding gene. This implies that LipR and Ni^2+^ coregulate certain metabolic processes, providing a logical basis for Ni^2+^ as an effector for LipR.

Balancing the correlation between glucose metabolism and intracellular pH is required for all living cells. Our work reveals that *B. thuringiensis*, a strain that effectively copes with glucose stress, uses a LacI‐TF LipR to regulate the intracellular pH by repressing *gapN* expression. On the basis of the distribution of the LipR‐like protein, LipR‐like proteins are widely used by bacteria to control metabolism. This study will lead to a deeper understanding of the sophisticated regulation and balance of the complex metabolism in bacteria.

## MATERIALS AND METHODS

### Bacterial strains and growth conditions

The primers, plasmids, and strains used in this study are listed in Table S1. *B. thuringiensis* BMB171 and its derivative strains were cultured in nutrient‐rich LB media (g/l: NaCl 10.0, yeast extract 5.0, and tryptone 10.0), nutrient‐poor GYS media (g/l: glucose 1; yeast extract 2; K_2_HPO_4_·3H_2_O 0.655; (NH_4_)_2_SO_4_ 2; MgSO_4_·7H_2_O 0.041; MnSO_4_·H_2_O 0.0378; CaCl_2_ 0.08), or YS media (glucose‐free GYS) at 28°C. When needed, 2% glucose was added to LB medium, and 0.2% glucose, fructose, galactose, sucrose, or trehalose was added to YS medium. When strains harbored certain plasmids, they were supplemented with 25 μg/ml erythromycin (MedChem Express) or 300 μg/ml spectinomycin (Mei5bio).

*E. coli* BL21 strains were routinely grown in LB medium at 37°C with rotary agitation at 200 rpm. For the cultivation of strains carrying the pET‐28(a) vector, 50 μg/ml kanamycin was supplemented.

### Protein purification

The ORFs of genes *lipR*, *ccpA*, and *abrB* were cloned into plasmid pET‐28(a) to create vectors pET‐*lipR*, pET‐*ccpA*, and pET‐*abrB* carrying a His‐tag on the C‐terminus of proteins LipR, CcpA, and AbrB, respectively. These three plasmids were transformed into the *E. coli* BL21 strain for protein expression and purification. To express the LipR protein, overnight cultures of strain BL21 harboring plasmid pET‐*lipR* were diluted 1:100 in LB medium supplemented with 50 μg/ml kanamycin and 10% v/v glycerol. After cultivation at 37°C for 3–4 h, the cultures were induced to express the LipR protein by adding 0.5 mM isopropyl‐β‐d‐thiogalactopyranoside. Then, the cultures were incubated at 16°C overnight with rotary agitation at 160 rpm. Following induction, cultures were subjected to centrifugation to harvest bacterial cells. The obtained cells were resuspended in 10 ml of lysis buffer (25 mM Tris pH 8.5–9, 500 mM NaCl, 10% glycerol, 10 mM imidazole, 1 mM PMSF) and sonicated, and the lysate was then spun down at 14,000 rpm for 30 min. The supernatants were collected for the subsequent purification process. The LipR protein was purified using a Ni‐nitriloacetic acid affinity column. Briefly, the column was balanced twice with lysis buffer and then coincubated with the obtained supernatant at 4°C for 20 min. Next, the column was washed three times with washing buffer (25 mM Tris pH 8.5–9, 500 mM NaCl, 10% glycerol, and 50 mM imidazole). Then, the LipR protein was eluted using eluting buffer (25 mM Tris pH 8.5–9, 500 mM NaCl, 10% glycerol, 250 mM imidazole). Purified LipR proteins were dialyzed overnight in buffer (25 mM Tris pH 8.5–9, 150 mM NaCl, 10% glycerol) to remove imidazole and other ions. Finally, the protein samples were stored at −80°C. For the purification of the proteins CcpA and AbrB, the method was based on a previous study^45^.

### Electrophoretic mobility shift assay

DNA fragments were amplified using PCR from genomic DNA of strain BMB171 and purified using a Universal DNA Purification Kit (TIANGEN). For the Cy5‐ or FAM‐labeled DNA fragments, the 5′‐end of their forward primers was tagged with Cy5 or FAM, and these primers were obtained from GENEWIZ (Tianjin). Purified DNA fragments were incubated with increasing amounts of protein CcpA, LipR, or AbrB in a 10 μl reaction volume at 28°C for 25 min. The reaction volume of LipR contained alkaline binding buffer A (25 mM Tris pH 8.5–9, 150 mM NaCl, 10% glycerol), and the binding buffer of CcpA and AbrB consisted of 50 mM Tris–HCI (pH 7.5), 10 mM MgCl_2_, 1 mM dithiothreitol, and 100 mM NaCl. When needed, 500 ng of poly(dI‐dC) were added to the reaction volume to reduce nonspecific binding. After incubation, 3 μl of 50% v/v glycerol was added, and the total mixture was subjected to 6% native polyacrylamide gel electrophoresis at 150 V for 90 min in 0.5× Tris borate EDTA running buffer. Finally, the gel image was visualized.

### Determination of intracellular pH

Overnight cultures of strains BMB171 and Δ*lipR* were diluted (OD_600_ = 0.01) in LB or YS medium supplemented with or without glucose at 28°C for 4 h. Then, the intracellular pH of these cells was determined using 2′,7′‐bis‐(2‐carboxyethyl)‐5‐(and‐6)‐carboxyfluorescein, acetoxymethyl ester (BCECF‐AM) (Beyotime), an intracellular pH probe used to measure the intracellular pH of bacteria. A 20 μM BCECF‐AM was added to the cultures at 28°C for 60 min. A standard curve was constructed using BMB171 cells resuspended in 100 mM phosphate buffer with 20 mM sodium benzoate. The fluorescence intensities of the above samples were measured at an excitation wavelength of 440 nm (Exw_440_, pH‐insensitive) or 488 nm (Exw_488_, pH‐sensitive) and an emission wavelength of 535 nm. The intracellular pH was calculated from the ratios of Exw_488_ to Exw_440_ using the standard curve^6^.

### Statistical analyses

For the qRT‐PCR and β‐galactosidase activity assays, results were calculated using at least three biological repeats and shown as the mean ± SD. These data were subjected to one‐way analysis of variance using Student's *t* test. Significance thresholds were specified as “ns” (no significant difference, *p* > 0.05), *p < 0.05, **p < 0.01, and ***p < 0.001^46^.

## AUTHOR CONTRIBUTIONS

Xia Cai and Jun Cai designed the study. Xia Cai carried out most of the experimental work and analyzed the data. Xuelian Li constructed the plasmids. Jiaxin Qin conducted EMSA experiments. Taoxiong Yuan and Bing Yan conducted the dual‐plasmid system. Jun Cai and Xia Cai wrote the manuscript. All authors read and approved the manuscript.

## ETHICS STATEMENT

This article does not contain any studies with human participants or animals performed by any of the authors.

## CONFLICT OF INTERESTS

The authors declare no conflict of interests.

## Supporting information

Supporting information.

Supporting information.

Supporting information.

Supporting information.

Supporting information.

## Data Availability

The RNA‐seq data have been deposited in the BioProject database under the accession number PRJNA858480.
