# Supplementary material for: LipR functions as an intracellular pH regulator in Bacillus thuringiensis under glucose conditions
Source: mLife. 2023 Feb 11;2(1):58–72. doi: 10.1002/mlf2.12055 (PMC10989752; doi:10.1002/mlf2.12055)
Supplement: Supplementary file 1 — Supporting information. [file MLF2-2-58-s004.doc]

**Supplementary Material for**

**“LipR functions as an intracellular pH regulator in *Bacillus thuringiensis* under glucose conditions”**

Xia Cai^1,2^, Jiaxin Qin^1^, Xuelian Li^1^, Taoxiong Yuan^1^, Bing Yan^1^, Jun Cai^1,3,4^,*

^1^ Department of Microbiology, College of Life Sciences, Nankai University, Tianjin, 300071, China

^2^ School of Life Sciences and Engineering, Lanzhou University of Technology, Lanzhou, 730050, China

^3^ Key Laboratory of Molecular Microbiology and Technology, Ministry of Education, Tianjin, 300071, China

^4^ Tianjin Key Laboratory of Microbial Functional Genomics, Tianjin, 300071, China

* **Corresponding author**: Jun Cai. Tel: +86 13920830631; **Email**: caijun@nankai.edu.cn

**Supplementary Materials and Methods**

**Plasmid construction**

For plasmids with pHT1K as the backbone: the promoter region of gene *lipR* was obtained using PCR with corresponding primer pairs and cloned into plasmid pHT1K via the *Nco* I - *Bam*H I sites to generate novel plasmid pB-P*lipR*. In this plasmid, the transcription of the reporter gene *lacZ* depends on the P*lipR* promoter, whereas the translation of *lacZ* depends on the ribosome binding site (RBS) of the *lacZ* gene.

The entire *yhfH* and *lipR* genes containing their promoter, encoding region, and terminator were generated using PCR and inserted into plasmid pHT1K-Δ*lacZ* via the *Nco* I - *Mlu* I sites to construct plasmids pB-OE*yhfH* and pB-OE*lipR*, respectively. Compared with plasmid pB-OE*yhfH*, plasmid pB-OE*yhfH*^M^ carries a mutated start codon (TAG) of the *yhfH* ORF; thus, it only produces RNA product YhfH and no polypeptide product. In plasmid pHT1K-Δ*lacZ*, the *lacZ* gene was deleted, and a *Mlu* I site was inserted into *Nco* I and *Kpn* I sites, the region from *Mlu* I to *Kpn* I site comprises a typical terminator.

For plasmids with pHT304 as the backbone: the DNA fragments containing the promoter P*lipR* and the *lipR* ORF with deleted stop codon TAG were ligated into plasmid pHT304 via a *Sac* I – *Kpn* I sites to generate plasmid pW-*lipR*. The LipR proteins encoded by plasmid pW-*lipR* were labeled with a Flag-tail.

**Determination of β-galactosidase activity**

The strains harboring the plasmid pB-P*lipR* or pB-P*yhfH* were cultured in the GYS or LB medium, respectively. The cultures at the designated time were collected and then used to detect the β-galactosidase activity as previously described^1^.

**Construction of mutant strains Δ*yhfH* and Δ*lipR***

The upstream and downstream DNA regions of the genes *yhfH* and *lipR* were obtained using PCR and ligated into the vector pRP1028 via *Sma* I - *Mlu* I and *Mlu* I - *Bam*H I sites to create plasmids pRP-UD*yhfH* and pRP-UD*lipR*, respectively. These two plasmids were electroporated into strain BMB171 for constructing strains Δ*yhfH* and Δ*lipR*. The detailed procedures were performed as previously described^2^. The mutant strains Δ*yhfH* and Δ*lipR* were verified using PCR and sequencing (GENEWIZ).

**RNA extraction and qRT-PCR**

According to the manufacturer's instructions, total RNA was extracted from BMB171 or its derivative strains using RNAiso Plus (TaKaRa). The obtained total RNA was treated with DNase I and then reverse transcribed as cDNA using a PrimeScript^TM^ RT reagent Kit with gDNA Eraser (TaKaRa). The synthesized cDNA was served as the DNA template for performing quantitative real-time PCR (qPCR) using TB Green® Premix Ex Taq™ II (Tli RNaseH Plus). The 16S rRNA was used as an internal control^3^.

**5’-Rapid amplification of cDNA ends (5’-RACE)**

The transcription start site (TSS) of the *lipR* gene was identified using 5’-RACE as previously described^3^. Briefly, the 3’-end of cDNAs were labeled with poly(dA) using terminal deoxynucleotidyl transferase (Takara). The tailed cDNA was used as a template to amplify target DNA fragments using primers *lipR*-R and 5’-RACE-F. The amplified DNA fragments were purified, ligated into the pMD19-T vector (Takara), and sequenced (GENEWIZ).

**Western blotting**

Total bacterial proteins were separated using SDS-PAGE electrophoresis and transferred onto a PVDF membrane. The detailed procedures were described in a previous study^4^. The LipR protein labeled with a Flag tail was probed with the primary antibody DYKDDDDK Tag (D6W5B) Rabbit mAb (Cell Signaling Technology) and the secondary antibody HRP Goat Anti-Rabbit IgG (H+L) (ABclonal). EF-Tu was used as a loading control and detected with an anti-eEF1A1/EFTu primary antibody (Abcam) and an HRP Goat Anti-Mouse IgG (H+L) secondary antibody (ABclonal)^1^.

**Construction of the dual-plasmids system**

Two compatible plasmids, pBMB2062 and pHT1K, were employed to construct a dual-plasmids system. Plasmid pBMB2062 was used to create sRNA-expressing vectors. The DNA fragments carrying the entire *yhfH* gene were amplified using PCR and then recombined into plasmid pBMB2062 digested by the *Bam*H I and *Sal* I. In this plasmid, the transcription and translation of the *yhfH* gene depend on its promoter and RBS.

Plasmid pHT1K is applicable for expressing the target gene translationally fused to the reporter gene *lacZ*. The region of the *lipR* gene (from +691 to +1011 of the ORF) that is entirely complementary to RNA YhfH was inserted into plasmid pHT1K via *Sal* I site to create vector pB-*lipR*-*lacZ*. In this plasmid, the 107 codons of the *lipR* gene were inserted after the first nine codons of the *lacZ* gene. The transcription of the fusion gene was controlled by a stable promoter P*kdp* derived from the *kdp* operon. The expression level of the fusion gene *lipR*-*lacZ* was determined by detecting β-galactosidase activity.

Plasmids pB-*lipR*-*lacZ* and pBMB2062-*yhfH* were co-transformed into strain BMB171 (Exg), empty plasmid pBMB2062 and pB-*lipR*-*lacZ* were co-transformed into strain BMB171 to construct control strain Ckg.

**Measurement of the growth curve**

Overnight cultures of strains BMB171, Δ*lipR*, and Δ*lipR*::*lipR* were diluted (OD_600_=0.01) in LB or YS medium at 28°C with rotary agitation at 200 rpm. When required, the YS medium was supplemented with 0.2% glucose, fructose, galactose, sucrose, or trehalose, and the LB medium was supplemented with 2% glucose. The optical density at 600 nm of these cultures was tested at different time points, and data were used to construct the growth curve.

**DNase I footprinting**

The precise binding sequence of LipR in the promoter P*lipR* was determined using DNase I footprinting. Firstly, the 190 ng FAM-labeled DNA fragments containing the P*lipR* promoter were incubated with 0 or 0.415 μg LipR protein in buffer A at 28°C for 25 min. After incubation, 0.26 U DNase-I (NEB) was added to the mixture to digest DNA fragments for 3.5 min. Then, the reaction was terminated at 65°C for 10 min. Finally, the digested DNA fragments were assayed with an ABI 3730XL DNA analyzer (GENEWIZ) and analyzed using the Peak Scanner software (Applied Biosystems).

**Bioinformatic analysis**

The -10 and -35 boxes of the promoter P*lipR* were analyzed using Softberry (<http://linux1.softberry.com/berry.phtml?topic=bprom&group=programs&subgroup=gfindb>), the potential binding sites of regulators CcpA and AbrB were scanned using DBTBS (http://dbtbs.hgc.jp/). The binding motif for LipR was predicted using MEME (<https://meme-suite.org/meme/tools/meme>), and binding sites of LipR embedded in the BMB171 genome were searched using FIMO (<https://meme-suite.org/meme/tools/fimo>).

**Supplementary Figure 1**

**
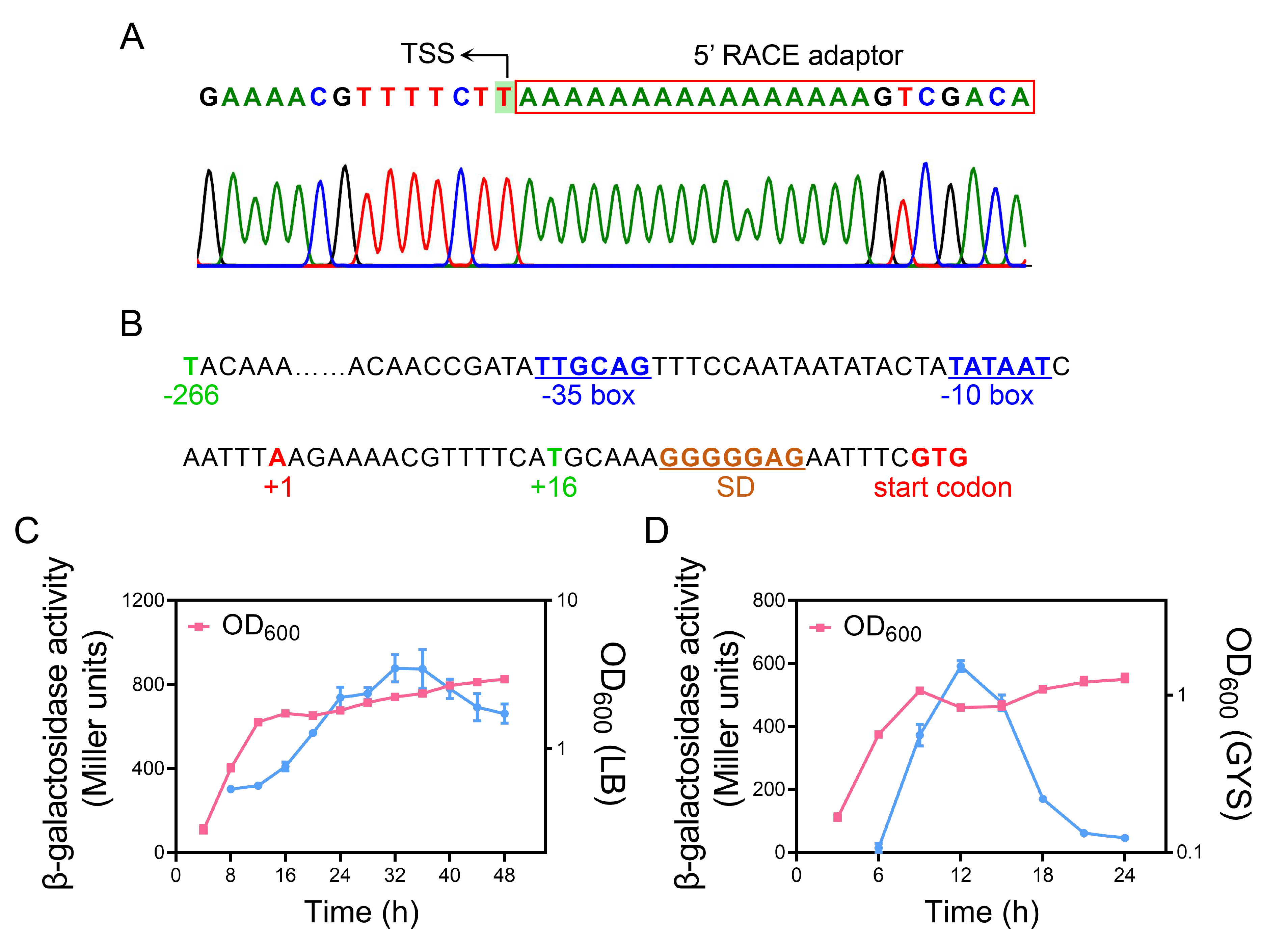
**

**Fig.S1** Mapping the transcription start site (TSS) of the *lipR* gene and characterizing the expression phase of its promoter P*lipR*. (A) Identification of the TSS of the *lipR* gene using 5’-RACE. The 5′-RACE adaptor (boxed in red) along with the *lipR* transcript after DNA sequencing are shown. The TSS is shaded in green. (B) The sequence of the promoter (-266 to +16) and Shine-Dalgarno (SD) of the *lipR* gene. The −35 box and −10 box are underlined and shown in blue, the TSS (A, +1) and start codon are depicted in red, and the SD is marked in brown. (C and D) Characterization of the expression phase of the P*lipR* promoter. The β-galactosidase activities of the strain BMB171/pB-P*lipR* were measured in LB (C) and GYS (D) media. These data are calculated from four biologically independent replicates and shown as the mean ± SD.

**Supplementary Figure 2**


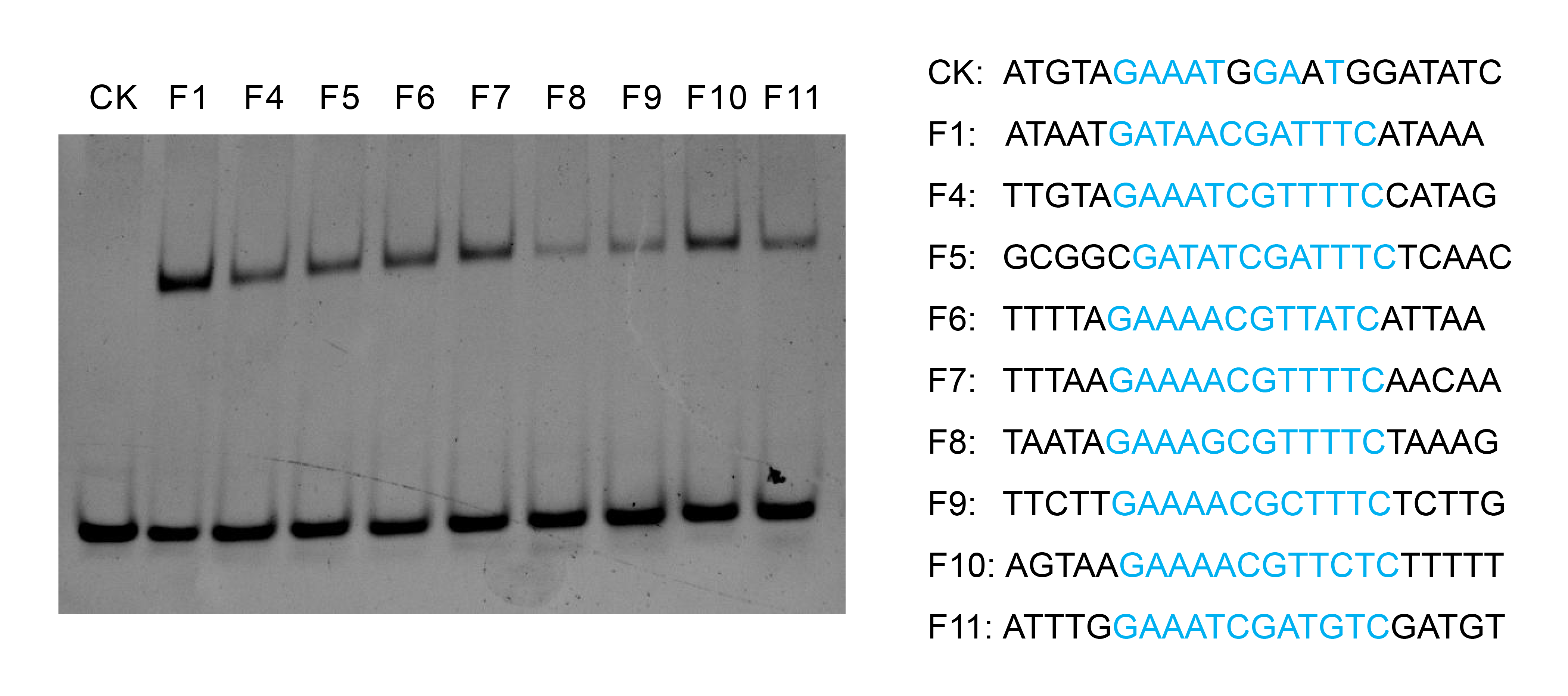


**Fig.S2** The binding of LipR protein with scanned potential binding sequences tested by EMSA.

**Supplementary Figure 3**


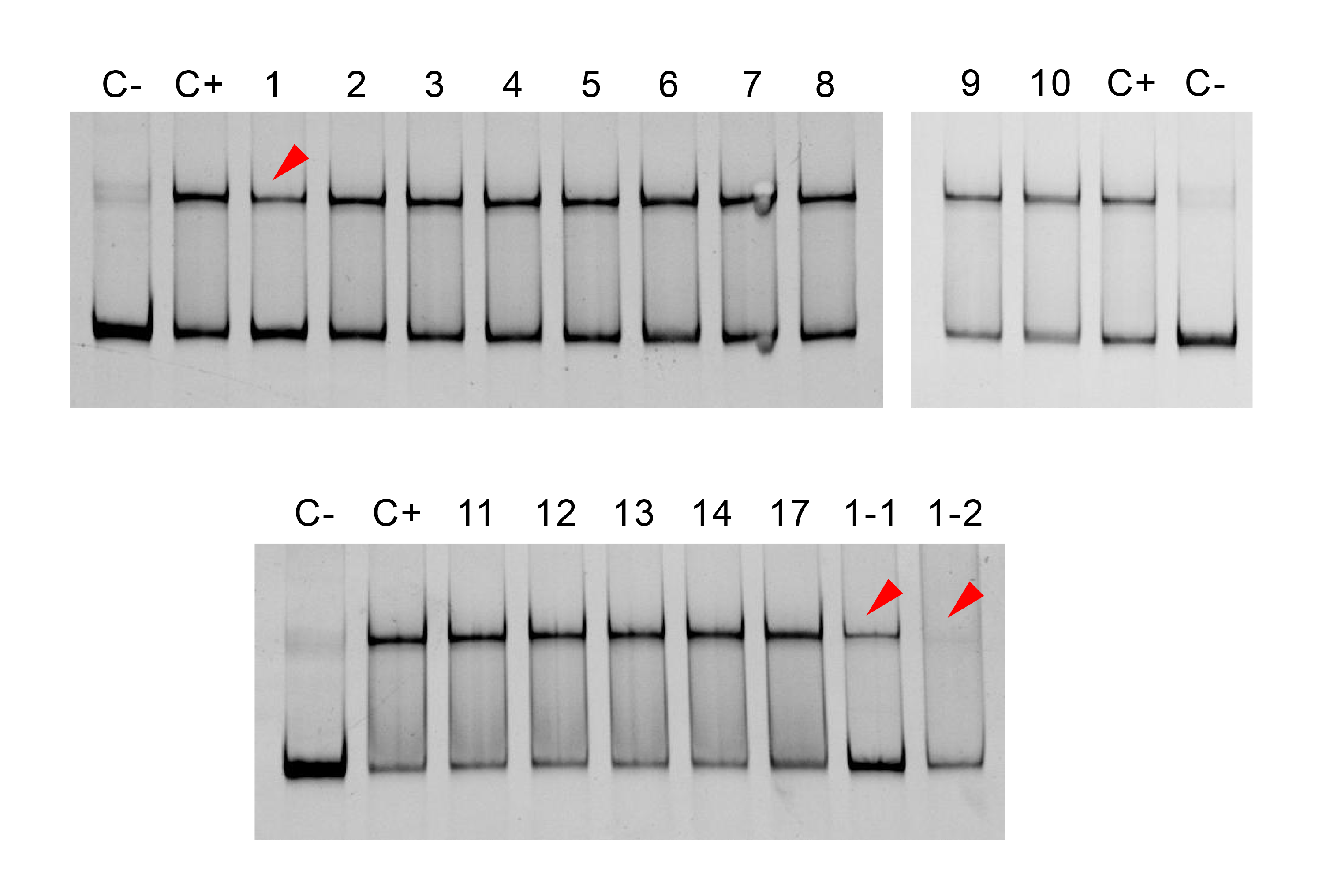


**Fig.S3** The effect of various sugars and intermediate metabolites on the binding of LipR protein with P*rppA* tested by EMSA. 1, pyruvate; 2, fructose-1, 6-bisphosphate; 3, glucose; 4, fructose; 5, xylose; 6, arabinose; 7, galactose; 8, maltose; 9, trehalose; 10, sucrose; 11, cellobiose; 12, mannose; 13, lactose; 14, glucose-6-phosphate; 17, phosphoenolpyruvate. C-, free LipR protein; C+, added LipR protein.

**Supplementary Figure 4**

**
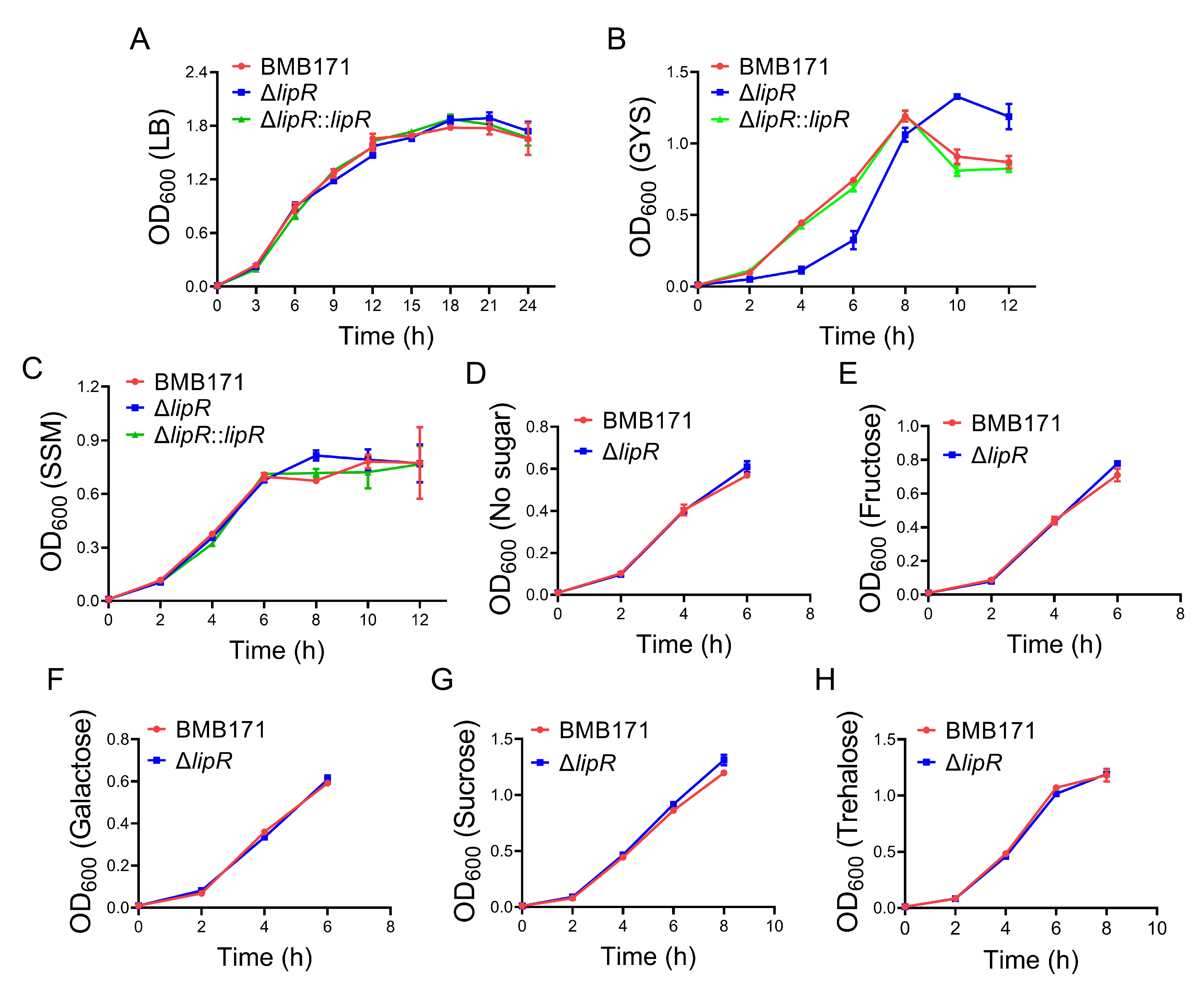
**

**Fig.S4** The cell growth of mutant strain Δ*lipR* is inhibited by glucose. (A-C) The growth curve of strains BMB171, Δ*lipR*, and Δ*lipR*::*lipR* in the LB, GYS, and SSM media. (D) The growth curve of strains BMB171 and Δ*lipR* in YS medium. (E-H) The growth curve of strains BMB171 and Δ*lipR* in YS medium with the addition of 0.2% fructose, galactose, sucrose, or trehalose, respectively. The results are calculated from four biologically independent replicates and are shown as the mean ± SD.

**Supplementary Figure 5**

**Fig.S5** The cell growth of strains BMB171, Δ*lipR*, and Δ*lipR*::*lipR* in YS medium with 0, 0.1%, and 0.2% glucose.

**Supplementary Figure 6**


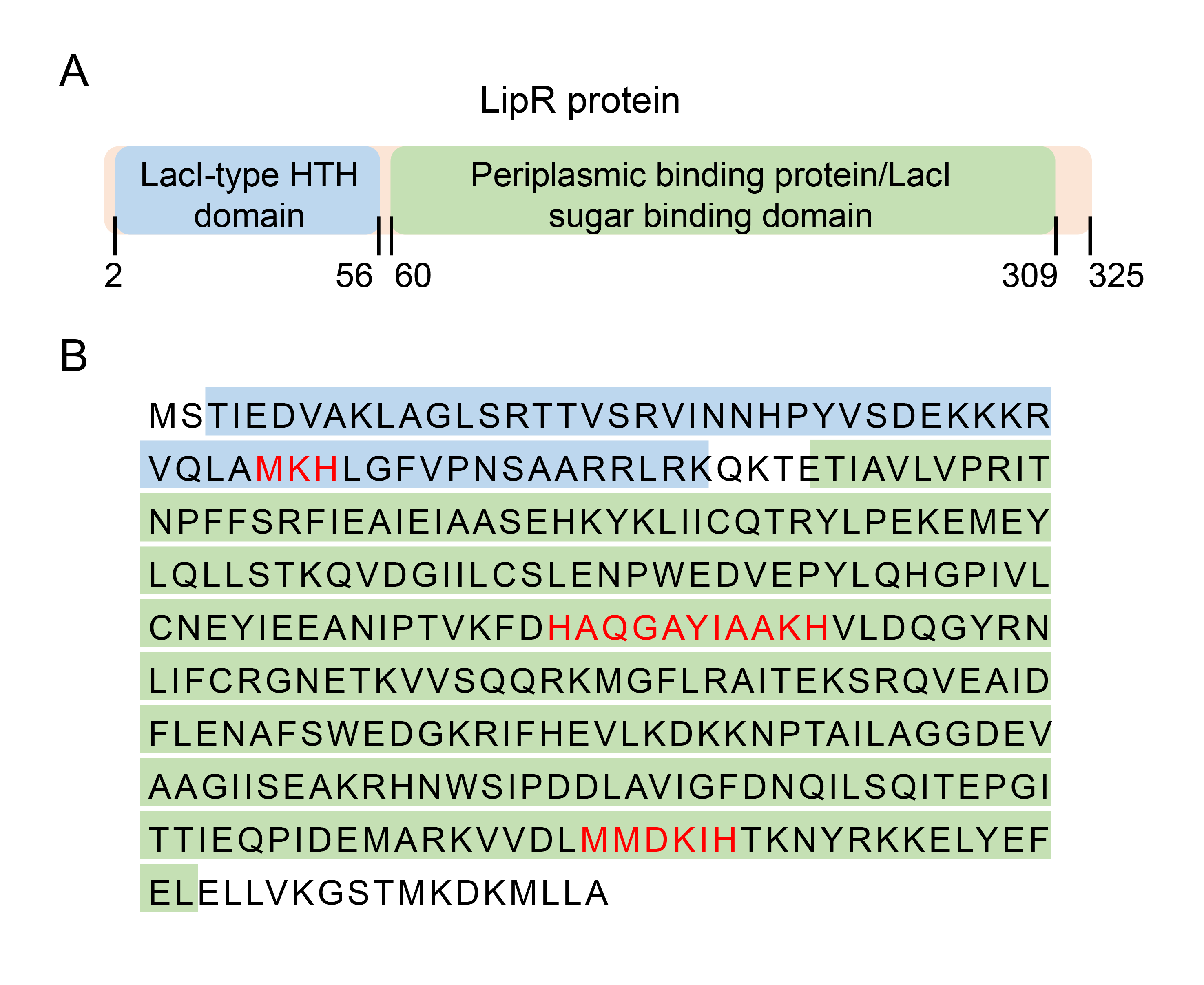


**Fig.S6** The predicted domain composition of LipR protein and potential Ni^2+^ binding sites embedded in it. (A) The domain structure of LipR protein was predicted using the InterPro database. The LacI-type HTH domain is depicted by a rounded light blue rectangle, and the Periplasmic binding protein/LacI sugar binding domain is shown by a green rectangle. In addition to these two domains, the other amino acids are drawn by the light pink rectangle. (B) Three potential binding sites of Ni^2+^ are embedded in LipR protein and shown in red.

Table S1 Primers, plasmids, and strains used in the study

| **Primers** | **Sequence (5’-3’)** | **Source** |
| --- | --- | --- |
| P*lipR*-R (*Bam*H I) | GGGGATCCATGAAAACGTTTTCTTAAATTGATT | In this study |
| P*lipR*-F (*Nco* I) | GGGccatggtacaaacgtatgggctttgtaga | In this study |
| *lipR*-R (*Mlu* I) | CGGacgcgtatgtgacgattgcgttcacgaaga | In this study |
| *yhfH*-F (*Nco* I) | GGGccatggactataatccatttcctaaactttttca | In this study |
| *yhfH*-R (*Mlu* I) | CGGacgcgtaaggagcatacatagctgcgaagca | In this study |
| *yhfH^M^-*F | agggattgacTAGatcgatcaaccaatggagtt | In this study |
| *yhfH^M^-*R | gttgatcgatCTAgtcaatccctccatttccat | In this study |
| 5'-RACE *lipR*-R | CACCCTAGAAACCGTTGTCC | In this study |
| Δ*lipR* U-F (*Hin*d III) | cagaagcttgtacgaggtgattcaatgatacggt | In this study |
| Δ*lipR* U-R | tttcgctgctggctaacgactaatgcaggggaatttatactgctt | In this study |
| Δ*lipR* D-F | taaattcccctgcattagtcgttagccagcagcgaaaaatgggct | In this study |
| Δ*lipR* D-R (*Mlu* I) | ccacgcgtgaaatggagggattgacatgatcga | In this study |
| Δ*yhfH* U-F (*Hin*d III) | cagaagcttggagcatacatagctgcgaagca | In this study |
| Δ*yhfH* U-R | tataatccatttcctaaactgaaaacttgtgcgcactgtgggaa | In this study |
| Δ*yhfH* D-F | cacagtgcgcacaagttttcagtttaggaaatggattatagtta | In this study |
| Δ*yhfH* D-R (*Mlu* I) | ccacgcgtatatgccctgcttttgcag | In this study |
| *lipR*-Flag-R (*Kpn* I) | GCTGGTACCggctaataacatcttatccttcatc | In this study |
| P*yifA*-*lipR*-F | aaactagggaggtttttttatgtcgactatagaggacgtggcg | In this study |
| P*yifA*-*lipR*-R | cgccacgtcctctatagtcgacataaaaaaacctccctagtttGGA | In this study |
| P*yifA*-F (*Sac* I) | TTCGAGCTCagggtatattgttttgggaagaga | In this study |
| pET-*lipR*-R (*Nco* I) | ATACCATGGGCgtgtcgactatagaggacgtggcga | In this study |
| pET-*lipR*-F (*Xho* I) | GTGCTCGAGggctaataacatcttatcct | In this study |
| P*lipR*-N_RocR_-F (*Nco* I) | GGGccatggTACAAACGTATGGGCTTTGTAGAAGAAGGCAGACAACGAAAGCGCGTTCGCTATGAAGATGGATCATATG | In this study |
| P*lipR*-N_AbrB_-R (*Bam*H I) | GGGGATCCATGAAAACGTTTTCTTAAATTGATTATATAGTATATTAGCGGACGCTGCAATATCGGTTGTTATG | In this study |
| P*lipR*-N_CcpA_-R (*Bam*H I) | GGGGATCCATGACGACGTGCTCTTAAATTGATTATA | In this study |
| *gapN*-F | ccgtacgggttgcgtaatga | In this study |
| *gapN*-R | gctttctctccattctccgt | In this study |
| RS12975-F | gacgcaaactctgaggtaga | In this study |
| RS12975-R | gccgctgcactagatcca | In this study |
| RS01340-F | gctccatagcaacattacgt | In this study |
| RS01340-R | gtcgcctaatatcatcatga | In this study |
| RS01690-F | gacgtttcacgttgtttgt | In this study |
| RS01690-R | ggcttcttgaatgggagct | In this study |
| RS22020-F | tgcgagaattagtagcgta | In this study |
| RS22020-R | acagccatttctccaagcat | In this study |
| RT- *gapN*-F | CCATCATGCTTTCTCCGTGC | In this study |
| RT- *gapN*-R | CTTCCCTACAAGATCGCGCA | In this study |
| 2062-*yhfH*-F (*Sal* I) | CAGGTCGACacatcctacattgttaacta | In this study |
| 2062-*yhfH*-R (*Bam*H I) | ATCGGATCCaaggagcatacatagctgcg | In this study |
| dp-*lipR*-F (*Sal* I) | ACGTAAGATTACGGGTCGACttaaaggacaaaaaaaatcctaccg | In this study |
| dp-*lipR*-R (*Sal* I) | CCAGGGTTTTCCCGGTCGACggctaataacatcttatccttca | In this study |
| **Plasmids** | **Characterizes** | **Source** |
| pW-*lipR* | The promoter P*yifA* and the CDS of the *lipR* gene are co-ligated into vector pHT304 via *Sac* I and *Kpn* I sites. The expression of *lipR* is driven by promoter P*yifA* and a Flag tail is fused to the C terminus of LipR protein | In this study |
| pHT304 | A low copy number (with 4 copies per cell) *E. coli* and *B. thuringiensis* shuttle vector; Amp^R^, Erm^R^ | 5 |
| pB-P*lipR* | The promoter P*lipR* fused with *lacZ* in *Nco* I and *Bam*H I sites of the plasmid pHT1K, transformed into BMB171 | In this study |
| pB-P*lipR*-N_CcpA_ | Compared to plasmid pB-P*lipR*, the predicted CcpA binding site in the promoter P*lipR* is mutated in plasmid pB-P*lipR*-N_CcpA_ | In this study |
| pB-P*lipR*-N_AbrB_ | Compared to plasmid pB-P*lipR*, the predicted AbrB binding site in the promoter P*lipR* is mutated in plasmid pB-P*lipR*-N_AbrB_ | In this study |
| pB-P*lipR*-N_RocR_ | Compared to plasmid pB-P*lipR*, the predicted RocR binding site is mutated in plasmid pB-P*lipR*-N_RocR_ | In this study |
| pET-*lipR* | The ORF of *lipR* is inserted in vector pET-28(a) via *Nco* I and *Xho* I sites | In this study |
| pET-*abrB* | The ORF of *abrB* is inserted in vector pET-28(a) via *Nco* I and *Xho* I sites | In this study |
| pET-*ccpA* | The ORF of *ccpA* is inserted in vector pET-28(a) via *Nco* I and *Xho* I sites | In this study |
| pHT1K | *B. thuringiensis*-*E. coli* shuttle plasmid; Amp^R^ Erm^R^, pHT1K vector harboring the promoter-less *lacZ* gene, transformed into BMB171 and used for β-galactosidase activity | 3 |
| pHT1K-Δ*lacZ* | The plasmid pHT1K deleting the *lacZ* gene | In this study |
| pB-OE*yhfH* | The promoter P*yhfH* and *yhfH* are inserted in vector pHT1K-Δ*lacZ* via *Nco* I and *Mlu* I sites | In this study |
| pB-OE*yhfH*^M^ | Compared to plasmid OE-*yhfH*, the start codon ATG of *yhfH* was replaced with stop codon TAG in plasmid OE-*yhfH*^M^ | In this study |
| pB-OE*lipR* | The promoter P*lipR* and *lipR* are inserted in vector pHT1K-Δ*lacZ* via *Nco* I and *Mlu* I sites | In this study |
| pRP-*yhfH-*UD | The upstream and downstream DNA fragments of *yhfH* are ligated into vector pRP1028. This plasmid is used to knock out the *yhfH* | In this study |
| pRP-*lipR-*UD | The upstream and downstream DNA fragments of *lipR* are ligated into vector pRP1028. This plasmid is used to knock out the *lipR* | In this study |
| pRP1028 | *B. thuringiensis*-*E. coli* shuttle plasmid; Amp^R^ Erm^R^; containing *turbo-rfp* gene and an I-*Sce* I recognition site | 3 |
| pBMB2062 | A high copy number (with 175-200 copies per cell) *E. coli* and *B. thuringiensis* shuttle vector; Amp^R^, Spe^R^ | 5 |
| pBMB2062-*yhfH* | The promoter P*yhfH* and *yhfH* are inserted in vector pBMB2062 via *Sal* I and *Bam*H I sites | In this study |
| pB-*lipR*-*lacZ* | The region of *lipR* gene (from +691 to +1011 of the ORF) was ligated into the plasmid pHT1K via a *Sal* I site | In this study |
| **Strains** | **Characterizes** | **Source** |
| *E. coli* DH5α | F- Φ80*lacZ*ΔM15 Δ(*lacZYA-argF*) U169 *rec*A1 *end*A1 *hsd*R17(rk^-^, mk^+^) *pho*A *sup*E44 *thi*-1 *gyr*A96 *rel*A1 λ- | / |
| BMB171 | *B. thuringiensis* strain BMB171; an acrystalliferous mutant strain; high transformation frequency | / |
| Δ*yhfH* | The *yhfH* gene was deleted in *B. thuringiensis* BMB171 | In this study |
| Δ*yhfH*::*yhfH* | Strain Δ*yhfH* carrying the plasmid pB-OE*yhfH* | In this study |
| Δ*yhfH*::*yhfH*^M^ | Strain Δ*yhfH* carrying the plasmid pB-OE*yhfH*^M^ | In this study |
| Δ*lipR* | The *lipR* gene was deleted in *B. thuringiensis* BMB171 | In this study |
| Δ*lipR*::*lipR* | Strain Δ*lipR* carrying the plasmid pB-OE*lipR* | In this study |
| BMB171/pW-*lipR* | Strain BMB171 carrying the plasmid pW-*lipR* | In this study |
| Δ*yhfH*/pW-*lipR* | Strain Δ*yhfH* carrying the plasmid pW-*lipR* | In this study |
| Δ*yhfH*/pB-P*lipR* | Strain Δ*yhfH* carrying the plasmid pB-P*lipR* | In this study |
| BMB171/pB-P*lipR* | Strain BMB171 carrying the plasmid pB-P*lipR* | In this study |
| BMB171/pB-P*lipR*-N_CcpA_ | Strain BMB171 carrying the plasmid pB-P*lipR*-N_CcpA_ | In this study |
| BMB171/pB-P*lipR*-N_RocR_ | Strain BMB171 carrying the plasmid pB-P*lipR*-N_RocR_ | In this study |
| BMB171/pB-P*lipR*-N_AbrB_ | Strain BMB171 carrying the plasmid pB-P*lipR*-N_AbrB_ | In this study |
| BL21/pET-*lipR* | Strain BL21(DE3) carrying the plasmid pET-*lipR* | In this study |
| BL21/pET-*ccpA* | Strain BL21(DE3) carrying the plasmid pET-*ccpA* | In this study |
| BL21/pET-*abrB* | Strain BL21(DE3) carrying the plasmid pET-*abrB* | In this study |

Table S4 The direct target genes of the LipR regulator identified by the EMSA

| **Gene locus** | | **Description** | **Up/down** | **Fold-**  **change** |
| --- | --- | --- | --- | --- |
| **Gene locus** | **Old gene locus** |
| BMB171_RS05535 | BMB171_C0956 | LacI family transcription regulator (LipR) | Up | / |
| BMB171_RS04385 | BMB171_C0746 | NADP-dependent glyceraldehyde-3-phosphate dehydrogenase | Up | 4.34 |
| BMB171_RS01340 | BMB171_C0223 | rhomboid family intramembrane serine protease | Down | 0.152 |
| BMB171_RS22020 | / | Uncharacterized membrane protein | Down | 0.245 |
| BMB171_RS12975 | BMB171_C2358 | Uncharacterized protein | Down | 0.154 |
| BMB171_RS01690 | BMB171_C0264 | Uncharacterized protein | Down | 0.193 |

Table S2. BMB171 vs Δ*lipR* Up-regulated genes

Table S3. BMB171 vs Δ*lipR* Down-regulated genes

Table S5. Potential binding sites of LipR scanned with FIMO

Table S6. Genes involved in the Glycolysis-Glycogenesis pathway listed in KEGG

**References**

1 Cai X, Wang Q, Fang Y, Yao D, Zhan Y, An B, et al. Attenuator LRR - a regulatory tool for modulating gene expression in Gram-positive bacteria. Microb Biotechnol. 2021;14: 2538-2551.

2 Zheng C, Ma Y, Wang X, Xie Y, Ali MK and He J. Functional analysis of the sporulation-specific diadenylate cyclase CdaS in *Bacillus thuringiensis*. Front Microbiol. 2015;6: 908.

3 Cai X, Zhan Y, Cao Z, Yan B and Cai J. Expression of ribosomal protection protein RppA is regulated by a ribosome-dependent ribo-regulator and two mistranslation products. Environ Microbiol. 2021;23: 696-712.

4 Jiang K, Hou XY, Tan TT, Cao ZL, Mei SQ, Yan B, et al. Scavenger receptor-C acts as a receptor for *Bacillus thuringiensis* vegetative insecticidal protein Vip3Aa and mediates the internalization of Vip3Aa via endocytosis. PLoS Pathog. 2018;14: e1007347.

5 Peng D, Luo X, Zhang N, Guo S, Zheng J, Chen L, et al. Small RNA-mediated Cry toxin silencing allows *Bacillus thuringiensis* to evade *Caenorhabditis elegans* avoidance behavioral defenses. Nucleic Acids Res. 2018;46: 159-173.
